# Identification of an autophagy-related 12-lncRNA signature and evaluation of NFYC-AS1 as a pro-cancer factor in lung adenocarcinoma

**DOI:** 10.3389/fgene.2022.834935

**Published:** 2022-08-11

**Authors:** Fang Tong, Lifa Xu, Sheng Xu, Mingming Zhang

**Affiliations:** ^1^ Department of Medical Immunology, School of Medicine, Anhui University of Science and Technology, Anhui, China; ^2^ Anhui Province Engineering Laboratory of Occupational Health and Safety, Anhui University of Science and Technology, Anhui, China; ^3^ The First Affiliated Hospital, Anhui University of Science and Technology, Anhui, China; ^4^ Chongqing Key Laboratory of Traditional Chinese Medicine for Prevention and Cure of Metabolic Diseases, College of Traditional Chinese Medicine, Chongqing Medical University, Chongqing, China

**Keywords:** LUAD, LncRNA, long noncoding RNA, autophagy, risk signature, prognostic indicator, NFYC-AS1, BIRC6

## Abstract

**Objective:** To develop an autophagy-related lncRNA-based risk signature and corresponding nomogram to predict overall survival (OS) for LUAD patients and investigate the possible meaning of screened factors.

**Methods:** Differentially expressed lncRNAs and autophagy genes were screened between normal and LUAD tumor samples from the TCGA LUAD dataset. Univariate and multivariate Cox regression analyses were performed to construct the lncRNA-based risk signature and nomogram incorporating clinical information. Then, the accuracy and sensitivity were confirmed by the AUC of ROC curves in both training and validation cohorts. qPCR, immunoblot, shRNA, and ectopic expression were used to verify the positive regulation of NFYC-AS1 on BIRC6. CCK-8, immunofluorescence, and flow cytometry were used to confirm the influence of NFYC-AS1 on cell proliferation, autophagy, and apoptosis *via* BIRC6.

**Results:** A 12-lncRNA risk signature and a nomogram combining related clinical information were constructed. Furthermore, the abnormal increase of NFYC-AS1 may promote LUAD progression through the autophagy-related gene BIRC6.

**Conclusion:** 12-lncRNA signature may function as a predictive marker for LUAD patients, and NFYC-AS1 along with BIRC6 may function as carcinogenic factors in a combinatorial manner.

## Introduction

Lung cancer accounts for 12.7% of the newly diagnosed cancer patients and up to 22.4% of deaths caused by cancer in 2020, according to the National Cancer Institute of the United States. About 50% of lung cancer cases belong to the lung adenocarcinoma (LUAD) type. Evaluation at early stages may help identify patients with a high-risk re-occurrence rate, develop a specific treatment strategy, and improve the overall survival rate of patients. Thus, the development of meaningful prognostic markers and the construction of related risk evaluation are essential for the diagnosis and treatment of LUAD patients.

Long noncoding RNA (lncRNA) is defined as a class of transcripts longer than 200 nucleotides (bps) and not translated into proteins (Iyer et al., 2015). LncRNA has been recently found to have many biological functions such as transcriptional regulation, translation, epigenetic modification, and cell fate decision (Ulitsky and Bartel, 2013; Li and Izpisua Belmonte, 2015). LncRNA has been demonstrated to be the driver of carcinogenesis, cancer progression, and metastasis in various cancers (Hirata et al., 2015; Yue et al., 2016; Yin et al., 2018; Tamang et al., 2019). For example, lncRNA-DANCR is overexpressed in tumor initiation cells, identifying DANCR as a prognostic biomarker and therapeutic target for hepatocellular carcinoma treatment (Yuan et al., 2016). Regarding LUAD, LncRNAs have been linked to various processes such as epithelial–mesenchymal transition (EMT), angiogenesis, and metastasis (Peng et al., 2018; Guan et al., 2019; Wang et al., 2020a).

Autophagy is an evolutionarily highly conserved catabolic process in eukaryotic cells (Han et al., 2014). It maintains the intracellular homeostasis of the environment by degrading and recycling the cellular components *via* the formation of autophagosome vesicles to engulf dysfunctional cytoplasmic organelles and the formation of autolysosome to degrade the contents of vesicles in the end (Klionsky, 2007; Fulda and Kögel, 2015). Autophagy plays a dual-faced role in tumor progression. On the one hand, it provides essential circulating metabolic substrates for biomolecule synthesis and supports the survival of cancer cells (Fulda and Kögel, 2015). On the other hand, autophagy could interplay with apoptosis to induce the death of cancer cells, including LUAD cells (Gewirtz, 2014; Liu et al., 2017). In addition, it plays an even more complex role in drug resistance, which depends on the activation status of autophagy and cellular environments (Li et al., 2017). Recently, accumulating evidence has shown that lncRNAs regulate the autophagy network *via* transcriptional regulations of autophagy-related genes in LUAD cells (Wang et al., 2019). Given the relevance of autophagy and lncRNAs in LUAD initiation and progression, this study focuses on investigating the expression patterns of autophagy-related lncRNAs from TCGA and Human Autophagy Database (HADb) to construct a useful risk signature to predict the prognosis of LUAD patients.

## Materials and methods

### Data collection and processing

**FIGURE 1:**
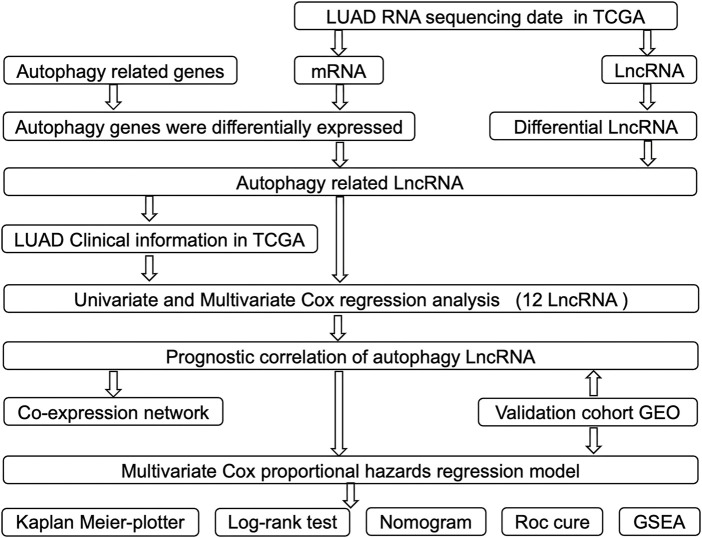
Flowchart of construction and validation of lncRNA risk signature and nomogram incorporating clinical parameters.

A detailed workflow for the construction of risk signature was developed, as shown in Figure 1. lncRNA and mRNA FPKM (fragments per kilobase of transcript per million fragments mapped) (level 3) sequencing profiles and associated clinical information of patients with LUAD were obtained from the TCGA data portal (https://tcgadata.nci.nih.gov/tcga/) before 1 November 2020. The GSE31210 microarray expression data were downloaded from the Gene Expression Omnibus (GEO) database, which included data of 226 LUAD samples, 20 normal tissue samples, and their associated clinical information. All autophagy-related genes were downloaded from the Human Autophagy Database (HADb) (http://www.autophagy.lu/). The “Limma” package of R software was used to screen differential autophagy-related gene expression and lncRNAs between adjacent and tumor tissues when | log_2_fold change (FC)| ≥ 0.5, and adjusted *p* < 0.05 was used as a filtering threshold. “Limma” package of R software was used to analyze the correlation between each differential lncRNA and every differential autophagy-related mRNA, and autophagy-related lncRNAs were screened when corFilter ≥0.3, and *p* < 0.05 was used as a filtering threshold.

### Identification of autophagy-related lncRNA prognostic signature

After the exclusion of LUAD patients with unavailable or insufficient follow-up clinical information, 458 LUAD patients were submitted for univariate Cox regression analysis. The “Survival” package of R software was used to perform the univariate regression analysis for the above lncRNAs, and prognosis-related lncRNAs were initially selected as candidates when *p* < 0.05. Next, the “Survival” package of the R software was used to perform multivariate COX regression analysis, and interdependent lncRNAs were identified as the risk signature, further validated by the Kaplan–Meier survival analysis. The risk score of each patient was based on a linear combination of lncRNA expression level (Exp) multiplied by a regression coefficient ( β ), represented as the following:

Risk score =∑i=1nExpi βi.

Then, based on the median risk score value, patients in the cohort were divided into high-risk and low-risk groups.

### Visualization of expression correlation network of autophagy-related genes and lncRNAs

After lncRNAs were identified as the risk signature, Cytoscape of version 3.6.1 was used to generate the expression correlations network between autophagy-related genes and lncRNAs when corFilter ≥ 0.3. The relations among high- or low-risk, autophagy-related genes and lncRNAs were also illustrated as a Sankey diagram to show the possible influence of autophagy-related lncRNAs on clinical outcomes.

### Risk signature assessment

After the risk signature was constructed, the Kaplan–Meier survival analysis was first used to evaluate the overall survival between high- and low-risk groups. Furthermore, to evaluate the predictive ability of the constructed risk signature, the time-dependent receiver operating characteristic (ROC) analyses were performed by the “Survival ROC” package of R software to test the signature’s sensitivity and specificity.

### Development and validation of a nomogram incorporating the lncRNA signature with clinical factors

Next, based on univariate and multivariate Cox regression results, a novel nomogram incorporating the 12-lncRNA signatures and related clinical factors was developed by the “rms” package of the R software. The predictive ability of the risk signature was further evaluated with the area under the curve (AUC) in the ROC analysis. “Caret,” “foreign,” and “survival” packages of R software were also used to randomly select half of the samples from the TCGA LUAD dataset as the internal cohort for validation. All the analyses were performed in the TCGA internal cohort and GSE dataset.

### Gene set enrichment analysis

Based on the median risk scores, 551 LUAD samples in the TCGA cohort were divided into two groups (high- and low-risk groups). Then, gene set enrichment analysis (GSEA) was performed to identify the significantly differentially expressed Kyoto Encyclopedia of Genes and Genomes (KEGG) pathways between the high- and low-risk groups using the Java-based GSEA software. A nominal (NOM) *p* < 0.05 was chosen as the cutoff value.

### Quantitative reverse transcription polymerase reaction assays for lung cancer belong to lung adenocarcinoma patient specimens and further validation in GSE40791 datasets

Ten paired LUAD tumor and adjacent normal tissues were obtained from patients of the First Affiliated Hospital of Anhui University of Science and Technology. All these patient tissues were transferred on ice and stored in liquid nitrogen. Total RNA was extracted from tissues with TRIzol reagent (Invitrogen, China) according to the manufacturer’s guide manual. Reverse transcription was performed according to the manufacturer’s instructions using the ReverTra Ace qPCR RT Kit (Toyobo, Japan). The SYBR Green Realtime PCR Master Mix (Toyobo) was used in the quantitative reverse transcription-polymerase chain reaction (qRT-PCR) experiment. Gene expression level was calculated with the 2-DDCt method. Primers in the qRT-PCR test are listed in Supplementary Table S1. Informed consent was signed by all the participants. The study was approved by the Ethics Committee of Chongqing Medical University. To further validate the expression patterns of these lncRNAs, we use GSE40791 datasets, which included 100 non-neoplastic (N) lung samples, and 69, 12, and 13 stages I, II, and II lung adenocarcinoma (AD) frozen tissues, respectively. The box plot is implemented by the “ggplot2” package of R software based on the Wilcoxon rank sum test.

### Cell culture, plasmid construction, and transfection

A549 cells were purchased from the Cell Bank of the Chinese Academy of Sciences (Shanghai, China) and cultured in Dulbecco’s MEM medium (Hyclone, Los Angeles, United States) supplemented with 10% fetal bovine serum and a 1% penicillin–streptomycin solution. All cells were incubated at 37°C in a humidified incubator containing 5% CO_2_. pEGFP-BIRC6 overexpression plasmid was constructed as previously described (Zhang et al., 2020a) by replacing it with the BIRC6 CDS region. For knockdown of NFYC-AS1, the specific shRNAs were synthesized and constructed into the same vector as previously described (Zhang et al., 2020a) by replacing targeted sequences with “CAC​CTG​TAA​TCC​CAG​CAC​TTT” (sh1) or “CAC​ACC​TGT​AAT​CCC​AGC​ATT” (sh2). Assayed cells were transfected with Lipofectamine® 2000 Reagent (Invitrogen) following the supplier’s instruction manual.

### Cell proliferation and apoptosis assays

Cells were seeded in 96-well plates at a density of 2,000 cells per well. The growth rate of cells was evaluated using the CCK-8 cell proliferation kit (Dojindo Laboratories, Kumamoto, Japan), according to the manufacturers’ instructions. OD detection at 450 nm was carried out by infinite 200Pro (Tecan). Cell apoptosis was analyzed using the Annexin V/7-AAD Apoptosis Detection kit (Keygen Biotech, Nanjing, China) on a CytoFLEX flow cytometer (Beckman, California, United States). Results were further analyzed by Flowjo 10.4.

### Western blotting, immunofluorescence assay, and immunohistochemistry

For immunoblotting experiment, cells were lysed in RIPA buffer (50 mM Tris (pH 7.4), 150 mM NaCl, 1% Triton X-100, 1% sodium deoxycholate, 0.1% SDS). An equal amount of protein was loaded and simultaneously subjected to electrophoresis in SDS-polyacrylamide gel and transferred to 0.22 μm pore-sized PVDF membranes (Roche, Basel, Switzerland). Membranes were briefly blocked with 5% skim milk and incubated with the primary antibodies overnight at 4°C, followed by incubation with the species-matched secondary antibody conjugated with HRP (Cell Signaling Technology, Danvers, United States) for 1 h at room temperature prior to chemiluminescence detection. For the immunofluorescence (IF) experiment, cells with less than 50% confluency were seeded and grown on coverslips overnight. Cells were fixed with 4% paraformaldehyde and permeabilized with Triton-100. After blocking in 5% goat serum, samples were incubated with the appropriately diluted primary and secondary antibodies. Eventually, cells were observed and photographed by fluorescence microscopy (Nikon C2). For the immunohistochemistry experiment, all tissues were fixed with 4% paraformaldehyde overnight at 4°C and then embedded in paraffin. The samples were subsequently sectioned into thin slices to be mounted on slides, followed by deparaffinization in xylene and rehydration through a series of ethanol–water solutions. Antigen retrieval was carried out by immersing the sections in citrate acid buffer with heating by a microwave oven. Slides were then blocked with 3% hydrogen peroxide to block nonspecific activity. After rinsing, slides were blocked with 5% BSA and then incubated with BIRC6 antibody overnight at 4°C. An immunohistochemical staining kit (BOSTER Biological Technology, Wuhan, China) was used for color development. Images were captured and confirmed by a professional pathologist under the microscope (Nikon, Tokyo, Japan). The color intensity of slides was divided into five grades (points) to score the BIRC6 expression level for statistical analysis. Antibodies used in the above experiments were as follows: β-actin (Cat No. 20536-1-AP, Proteintech, 1:1000), BIRC6 (ab19609, Abcam, 1:2000), Beclin 1 (Cat No. 11306-1-AP, Proteintech, 1:1000), SQSTM1/p62 (Cat No. 18420-1-AP, Proteintech, 1:1000), LC3B (Cat. No. NB600-1384, Novus Biologicals, 1:500), and BIRC6 (29760-1-AP, Proteintech, 1:200) for immunohistochemistry.

### Immunodeficient xenograft mouse tumor model

BALB/c-nude mice (4–5 weeks of age, 18–20g) were purchased from Beijing Vital River Laboratory Animal Technology Co., Ltd. (China). All experimental procedures were approved by the Institutional Animal Care and Use Committee of Chongqing Medical University. Briefly, cells were trypsinized, washed, and resuspended in phosphate-buffered saline at a density of 10^7^ cells/ml. A total of fifteen mice were inoculated subcutaneously with 5×10^6^ cells of A549 Vector cells, A549 NFYC-AS1 knockdown cells, and A549 NFYC-AS1 knockdown with BIRC6 overexpression cells, respectively. Mice were sacrificed 7 days after tumor cell implantation, and the tumors were removed and weighed. GraphPad Prism 8 and unpaired *t*-test were used for statistical analysis.

## Results

### Differential expression of autophagy-related genes and lncRNAs in lung cancer belong to lung adenocarcinoma patients

**FIGURE 2:**
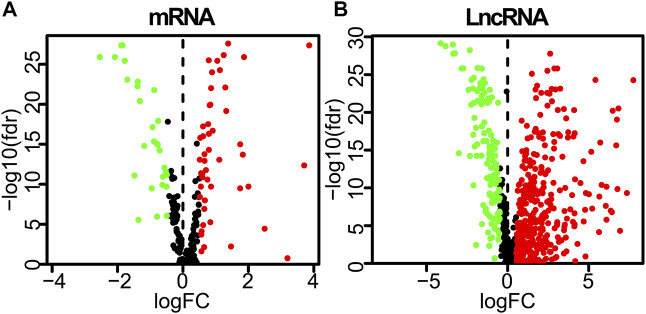
Differently expressed autophagy-related genes and lncRNAs based on the correlation coefficient | log2(FC)| ≥ 0.5 and adjusted *p* < 0.05. **(A)** Volcano plot of differently expressed autophagy-related genes. **(B)** Volcano plot of differently expressed lncRNAs between LUAD tumor and normal samples. FC, fold change.

A detailed data processing flowchart is shown in Figure 1. Clinical and diagnostic information of LUAD patients in the TCGA and validation cohorts are shown in Table 1. A total of 232 autophagy genes were collected from the HADb database, and we identified 76 differentially expressed autophagy genes between 497 tumor tissues and 54 normal tissues from TCGA at the criteria of | log_2_(FC)| ≥ 0.5 and an adjusted *p* < 0.05 (Figure 2A). A total of 507 differentially expressed lncRNAs were identified, including 373 upregulated and 134 downregulated lncRNAs (Figure 2B). Then, a total of 107 lncRNAs related to autophagy were screened using the “Limma” package of R software when the criteria were set to corFilte ≥ 0.3 and *p* < 0.05 (Supplementary File S1).

**TABLE 1:** Primers for the detection of lncRNAs expression in LUAD specimens.

Primer	Sequence
HCG18-F	TGA​AAG​TCG​ACG​AAG​AGG​GC
HCG18-R	ACT​AGT​CGG​AAG​TGA​CGT​GC
TMPO-AS1-F	GCA​GTT​TAA​AAG​GCG​CTG​GG
TMPO-AS1-R	CCC​GAT​TGT​AGA​GGT​CTG​GC
NFYC-AS1-F	CTT​CCC​TAT​CAC​CCT​CGC​AC
NFYC-AS1-R	GATCTCGAG CTCCCGAAAGG
LINC00996-F	CTC​CCA​TCT​TTT​CTG​CCG​GT
LINC00996-R	AGG​GGC​ATC​CAA​AGG​TCT​TG

### Identification of autophagy-related lncRNA prognostic signature for lung cancer belonging to lung adenocarcinoma

**FIGURE 3:**
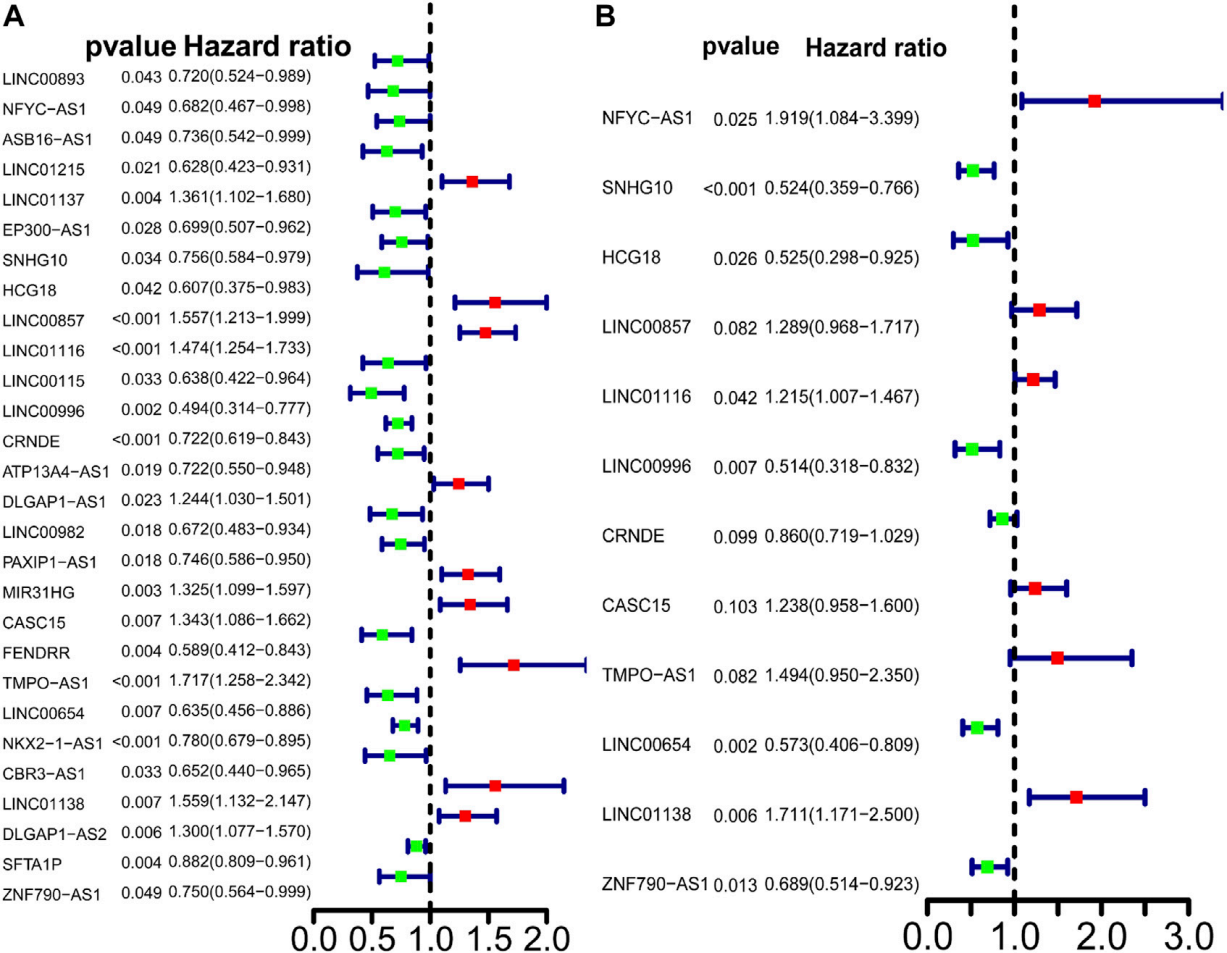
Predictive power of the differently expressed lncRNAs in LUAD patients. **(A)** Univariate Cox regression analysis of differently expressed autophagy-related lncRNAs. Cox analysis. **(B)** Multivariate Cox analysis of differently expressed autophagy-related lncRNAs.

**FIGURE 4:**
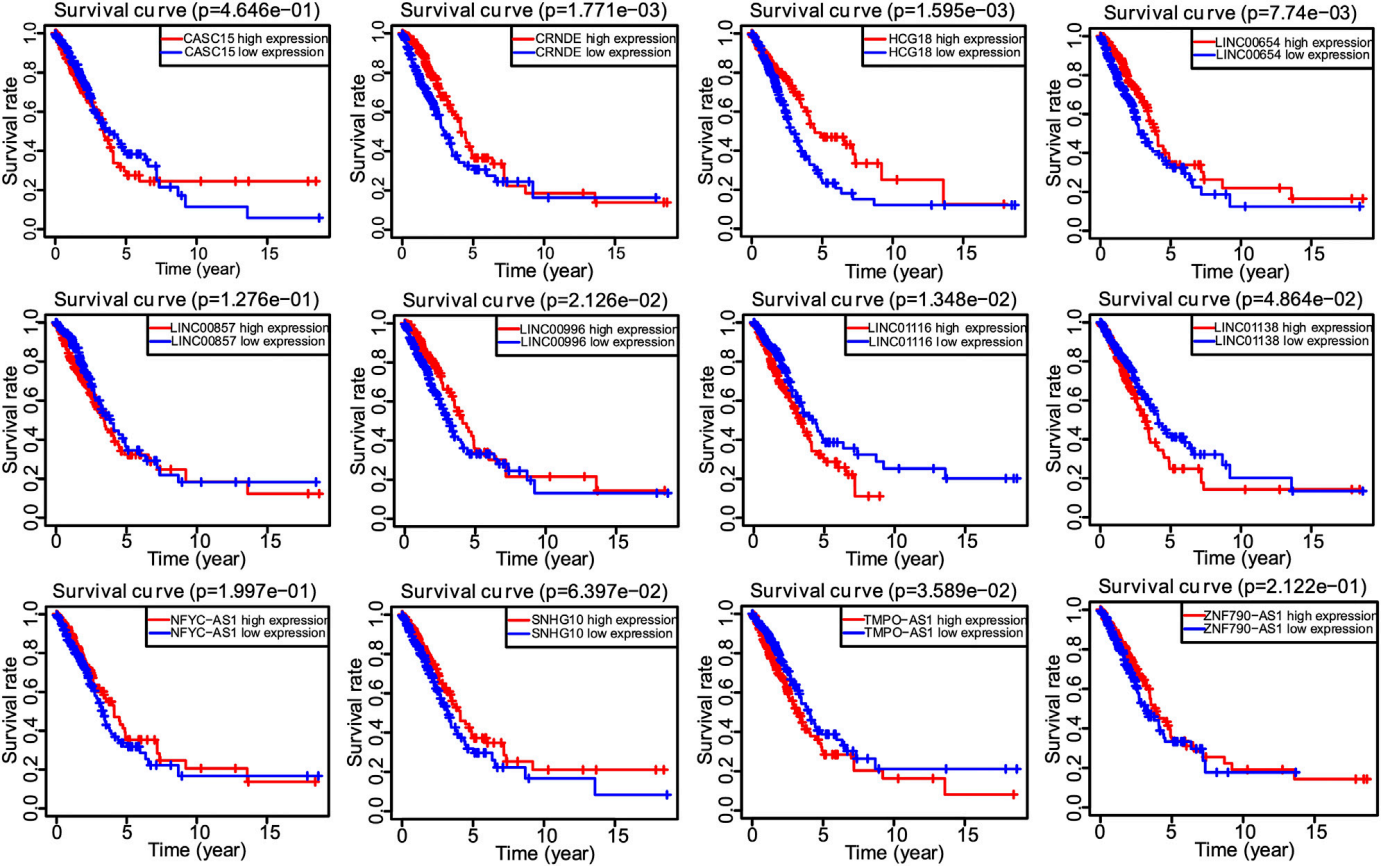
The Kaplan–Meier survival analysis of 12 screened lncRNAs.

**FIGURE 5:**
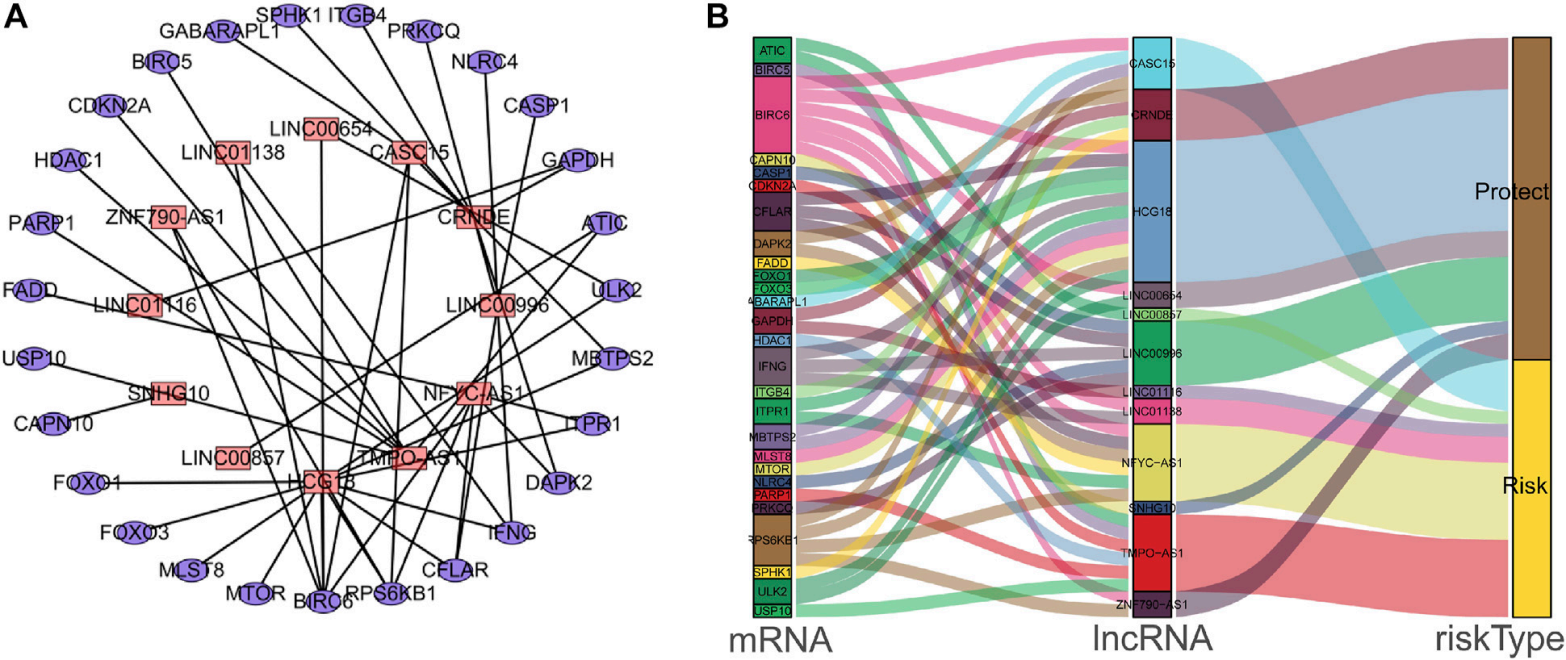
The interaction network of autophagy genes and OS-associated lncRNAs in LUAD patients. **(A)** The co-expression network of the autophagy-related mRNAs and lncRNAs was constructed and visualized using Cytoscape. **(B)** The co-expression network with risk-type information was visualized using the Sankey diagram.

Univariate Cox regression analysis identified 28 out of 107 lncRNAs as prognostic factors (Figure 3A). Next, multivariate Cox regression was performed and 12 lncRNAs were further found to be independent factors in predicting the prognosis of LUAD patients (Figure 3B). Results of the Kaplan–Meier survival analysis further confirmed the relevance between these 12 lncRNAs and OS (Figure 4). These 12 lncRNAs are NFYC-AS1, SNHG10, HCG18, LINC00857, LINC01116, LINC00996, CRNDE, CASC15, TMPO-AS1, LINC00654, LINC01138, and ZNF790-AS1. An expression correlation network was built on the 12 OS-associated lncRNAs and 16 corresponding associated autophagy-related genes, including FOXO1, FOXO3, MLST8, MTOR, BIRC6, RPS6KB1, CFLAR, IFNG, DAPK2, ITPR1, MBTPS2, ULK2, ATIC, GAPDH, CASP1, NLRC4, PRKCQ, ITGB4, SPHK1, GABARAPL1, BIRC5, CDKN2A, HDAC1, PARP1, FADD, USP10, and CAPN10 as shown in Figure 5.

### Overall survival-associated lncRNA-based risk signature development for lung cancer belonging to lung adenocarcinoma

**FIGURE 6:**
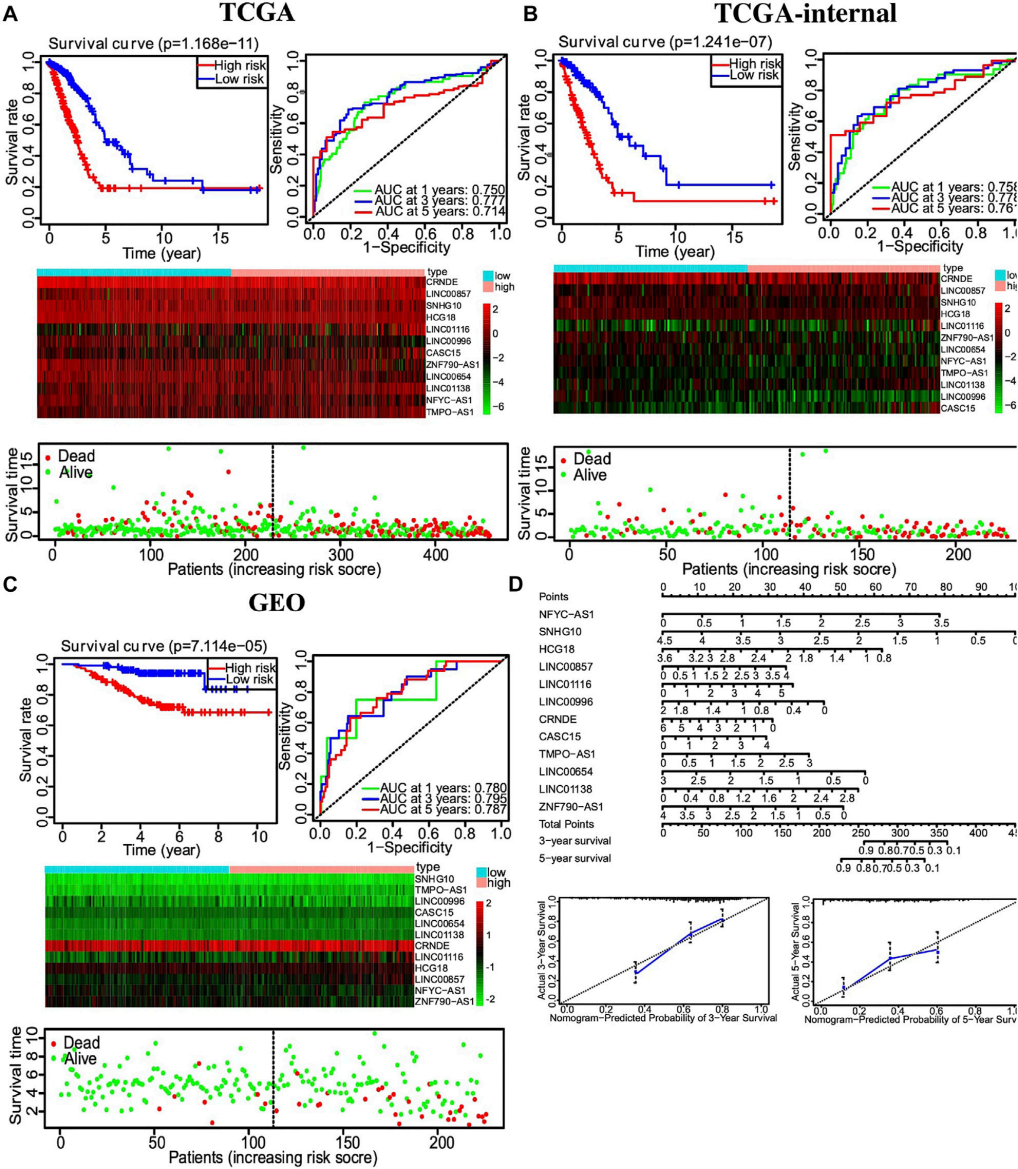
Autophagy-related lncRNA risk score analysis of LUAD patients. **(A)** Top left, Kaplan–Meier survival analysis of the high- and low-risk groups based on the median risk score. Top right, 1-, 3-, and 5-year AUC value of ROC analysis. Middle, heatmap of autophagy-related lncRNAs expression profiles in the low- and high-risk groups. Bottom, the scatterplot based on the survival status of each patient. **(B)** Similar results to 6A from the TCGA train dataset. **(C)** Similar results to 6A from the GSE validation dataset. **(D)** Nomogram of 12-lncRNA signature based on multivariate Cox proportional hazards regression analyses.

Multivariate Cox regression was used to develop a risk signature consisting of 12 autophagy-related lncRNAs with the “Survival” package of R to predict the OS of LUAD. Risk score = (0.651*EXP_NFYC-AS1) + (−0.645* EXP_ SNHG10) + (0.644*EXP_HCG18) + (0.254*EXP_LINC00857) + (0.194*EXP_LINC01116) + (−0.664*EXP_LINC00996) + (−0.150*EXP_CRNDE) + (0.213*EXP_CASC15) + (−0.401* EXP_TMPO-AS1) + (0.556*EXP_LINC00654) + (0.537*EXP_LINC01138) + (0.372*EXP_ZNF790-AS1). A total of 458 LUAD patients were divided into high- and low-risk groups based on the median risk score. Kaplan–Meier survival analysis showed a significant OS advantage of the low-risk group over the high-risk group, as shown in Figure 6A (*p* < 0.001). Moreover, more than 0.7 of 1-, 3-, and 5-year area under the curve (AUC) in receiver operating characteristic (ROC) analysis exhibited a satisfactory diagnostic performance (Figure 6A). We further validated this risk signature in both TCGA internal cohort and the GSE validation dataset. Once again, Kaplan–Meier survival analysis showed an improved OS in the low-risk group compared to the high-risk group, as shown in Figures 6B,C. 0.7 of 1-, 3-, and 5-year AUC in ROC analysis also showed a fair sensitivity and specificity of this risk signature. Then, based on the multivariate Cox proportional hazards regression analyses, a nomogram including 12-lncRNA signatures was constructed by the ‘‘rms’’ package in R software to help predict the 3- or 5-year survival of LUAD patients (Figure 6D).

### Nomogram development and validation

**FIGURE 7:**
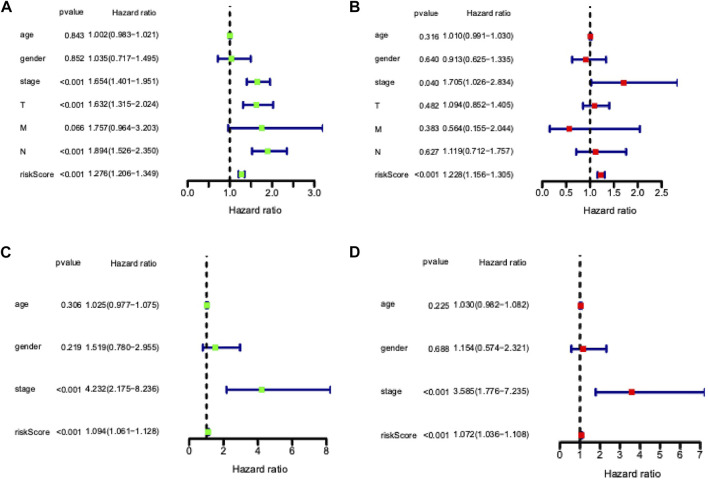
Independent prognostic ability evaluation for the constructed prognostic signature in LUAD. **(A)** Univariate Cox regression analysis of risk score and related clinical information regarding prognostic value in TCGA LUAD dataset. **(B)** Multivariate Cox analysis of risk model score and clinical features regarding prognostic value in the TCGA LUAD dataset. **(C)** Univariate Cox regression analysis of risk score and related clinical information regarding prognostic value in GSE dataset. **(D)** Multivariate Cox analysis of risk model score and clinical features regarding prognostic value in GSE dataset.

**FIGURE 8:**
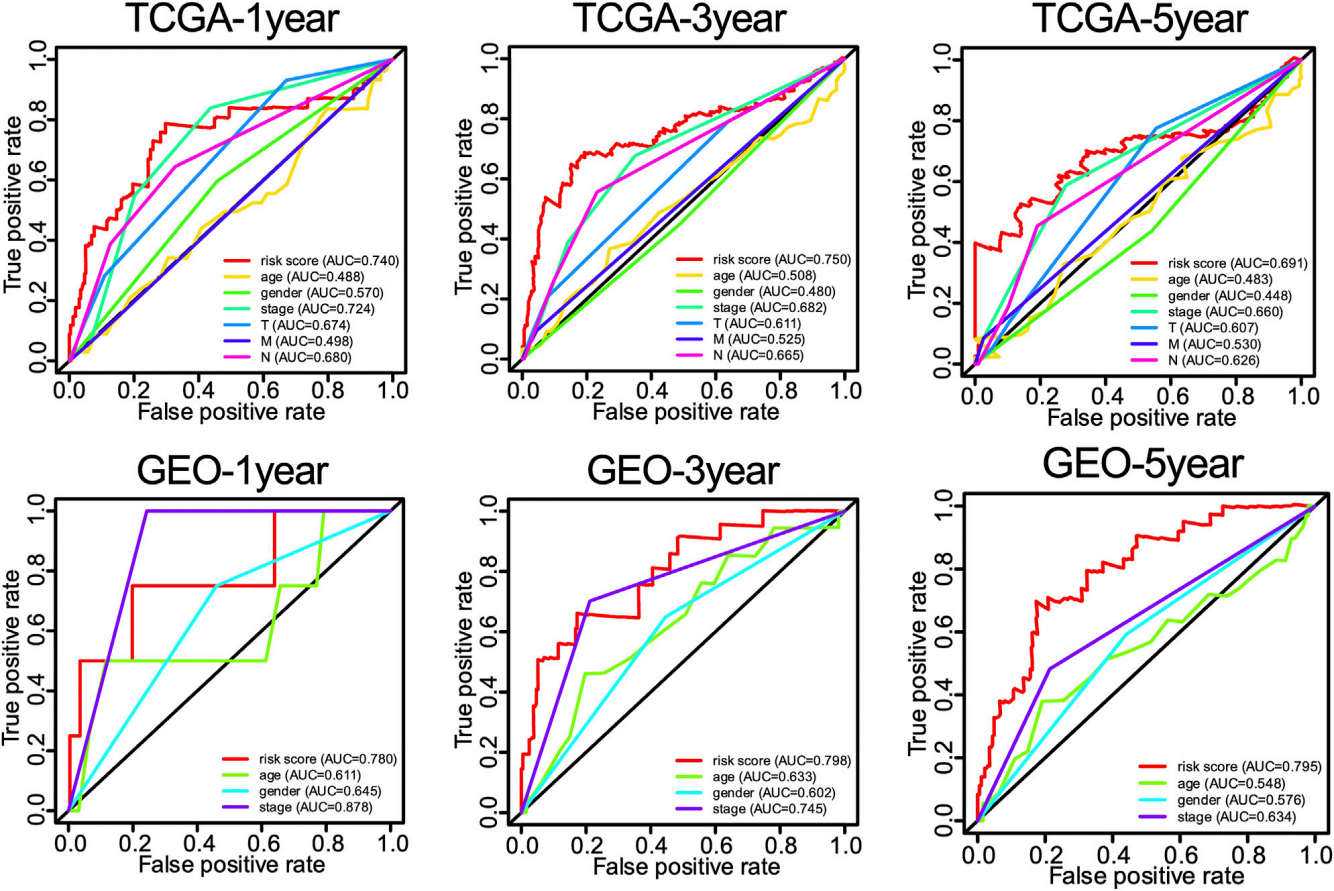
The 1-, 3-, and 5-year ROC curves of risk score and related clinical information of LUAD patients.

**FIGURE 9:**
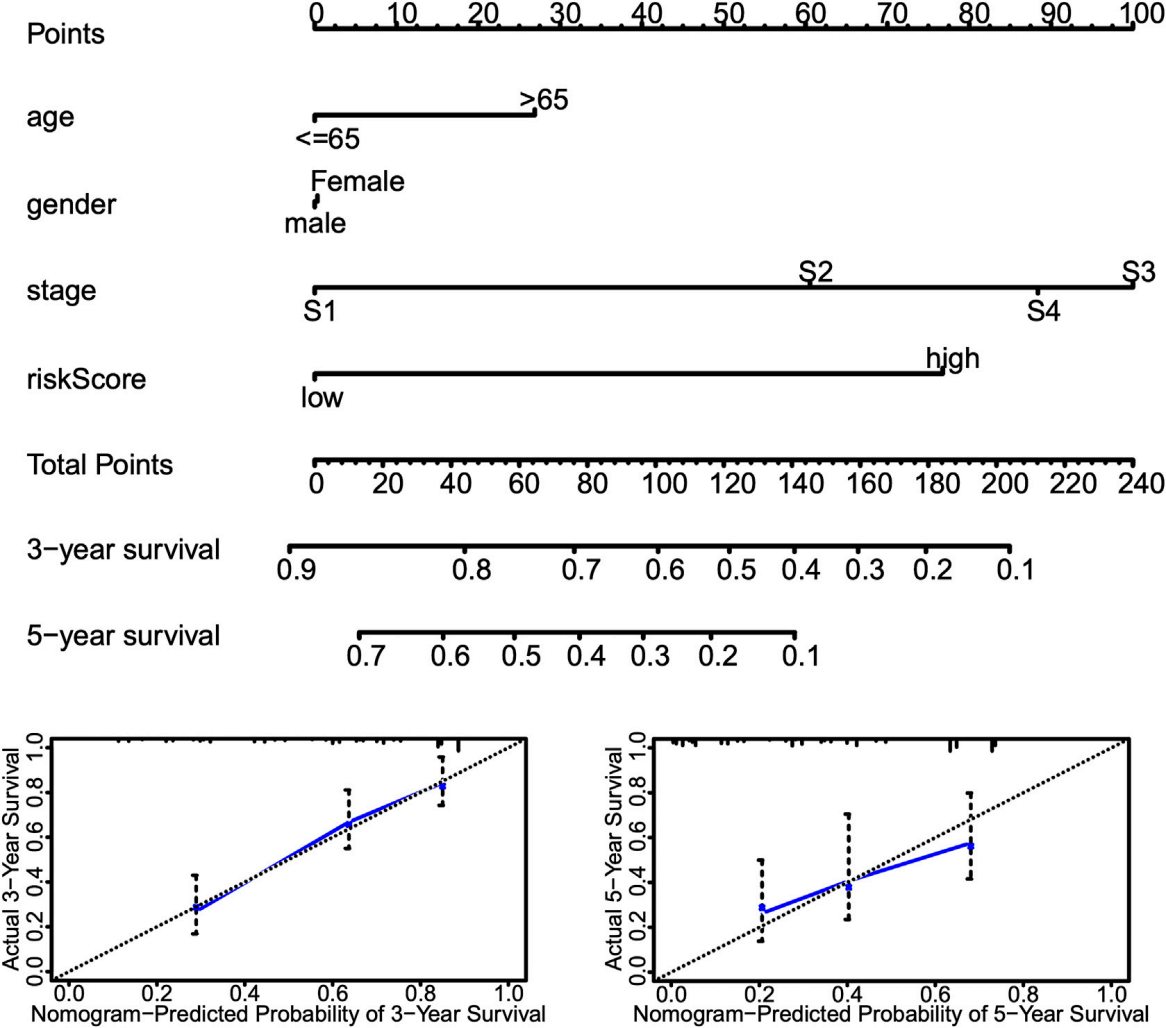
Nomogram incorporating risk score and other clinical information. Top, nomograms to predict 3- or 5-year OS of patients with LUAD. Bottom, calibration curves of the nomogram for 3- or 5-year OS prediction.

Univariate and multivariate Cox proportional hazards regression analyses were performed to evaluate the predictive value of different clinical parameters. Univariate Cox regression analysis showed that stage, T status, N status, and risk score were significantly associated with OS of LUAD patients in TCGA datasets, as shown in Figure 7A (*p* < 0.001). On the other hand, multivariate Cox regression analysis only showed that stage and risk score are the independent predictive factor of OS for LUAD patients (Figure 7B). In the GSE31210 dataset, univariate Cox regression analysis showed that the stage and risk score are significantly related to the OS of LUAD patients (Figure 7C). Multivariate Cox regression showed that stage and risk score are the independent predictive factors of OS (Figure 7D). Also, risk signature based on 12 lncRNA showed 1-, 3-, and 5-year AUC of above 0.69 in ROC analysis along with other clinical factors in both TCGA and GEO datasets (Figure 8). Then, we constructed a nomogram incorporating 12-lncRNA signatures and other clinical information by the ‘‘rms’’ package in R software to predict the 3- and 5-year survival of LUAD patients. The following calibration curve showed a satisfactory consistency between the predictive and observed results (Figure 9).

### Validation of the expression of lncRNAs in lung cancer belong to lung adenocarcinoma patient samples and GSE40791

**FIGURE 10:**
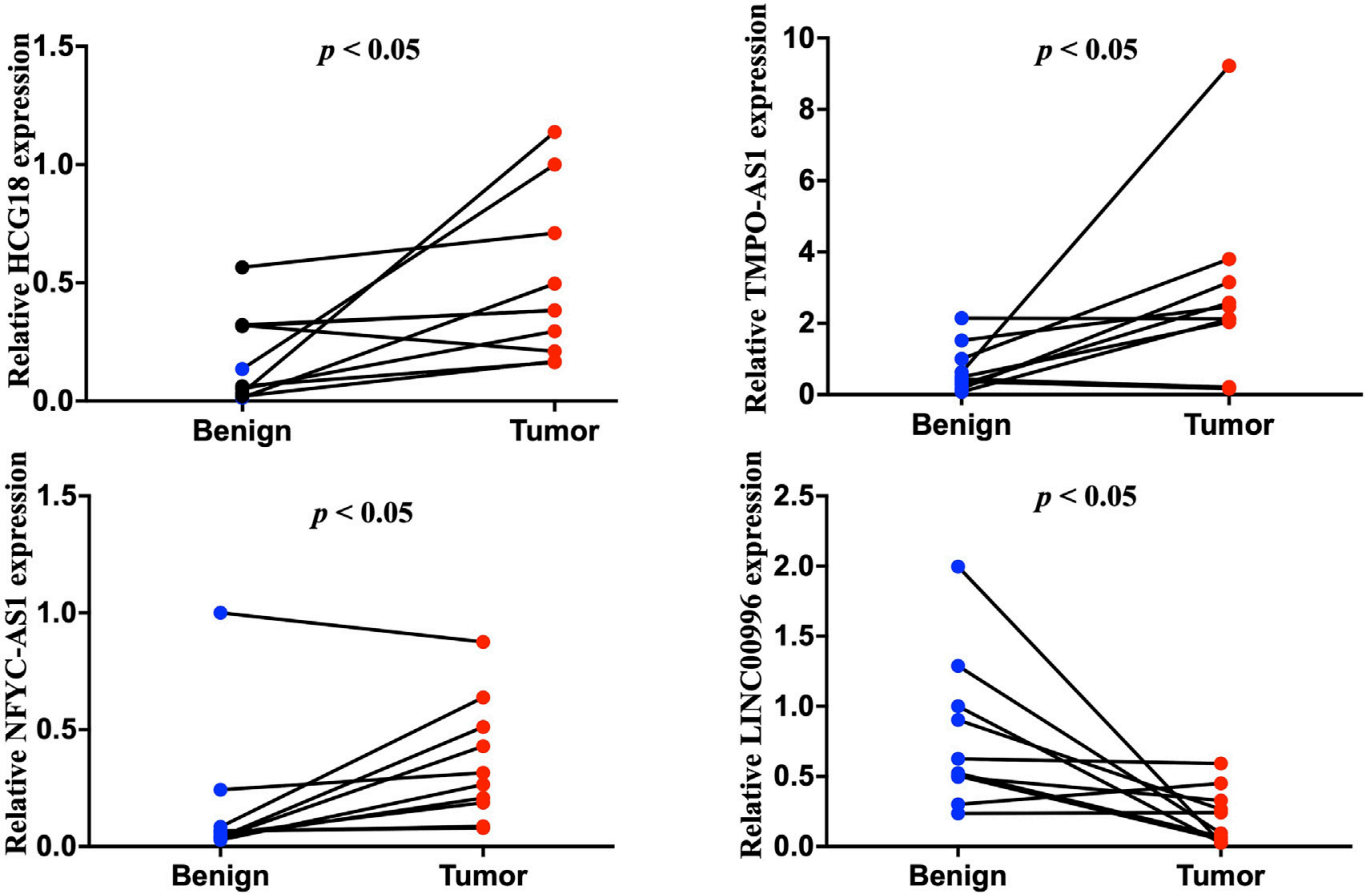
Quantitative reverse transcription polymerase reaction results of four lncRNAs, including HCG18, TMPO-AS1, NFYC-AS1, and LNC00996, from ten LUAD patients.

The expression of selected lncRNAs from our constructed signatures (HCG18, TMPO-AS1, NFYC-AS1, LNC00996) was investigated in 10 paired tumor and adjacent tissues from LUAD patients using qRT-PCR. The results indicated that HCG18, TMPO-AS1, and NFYC-AS1 were significantly upregulated in tumors compared with adjacent samples. In contrast, LNC00996 were significantly downregulated in tumors compared with adjacent samples (Figure 10) (*p* < 0.05). These results were consistent with the expression pattern from the TCGA LUAD dataset (Supplementary Figure S1). Also, results from the GSE40791 dataset, including 100 normal lung and 94 LUAD lung tissues, further validate the expression pattern of these lncRNAs (Supplementary Figure S2).

### lncRNA NFYC-AS1 promotes lung adenocarcinoma cell proliferation through BIRC6

**FIGURE 11:**
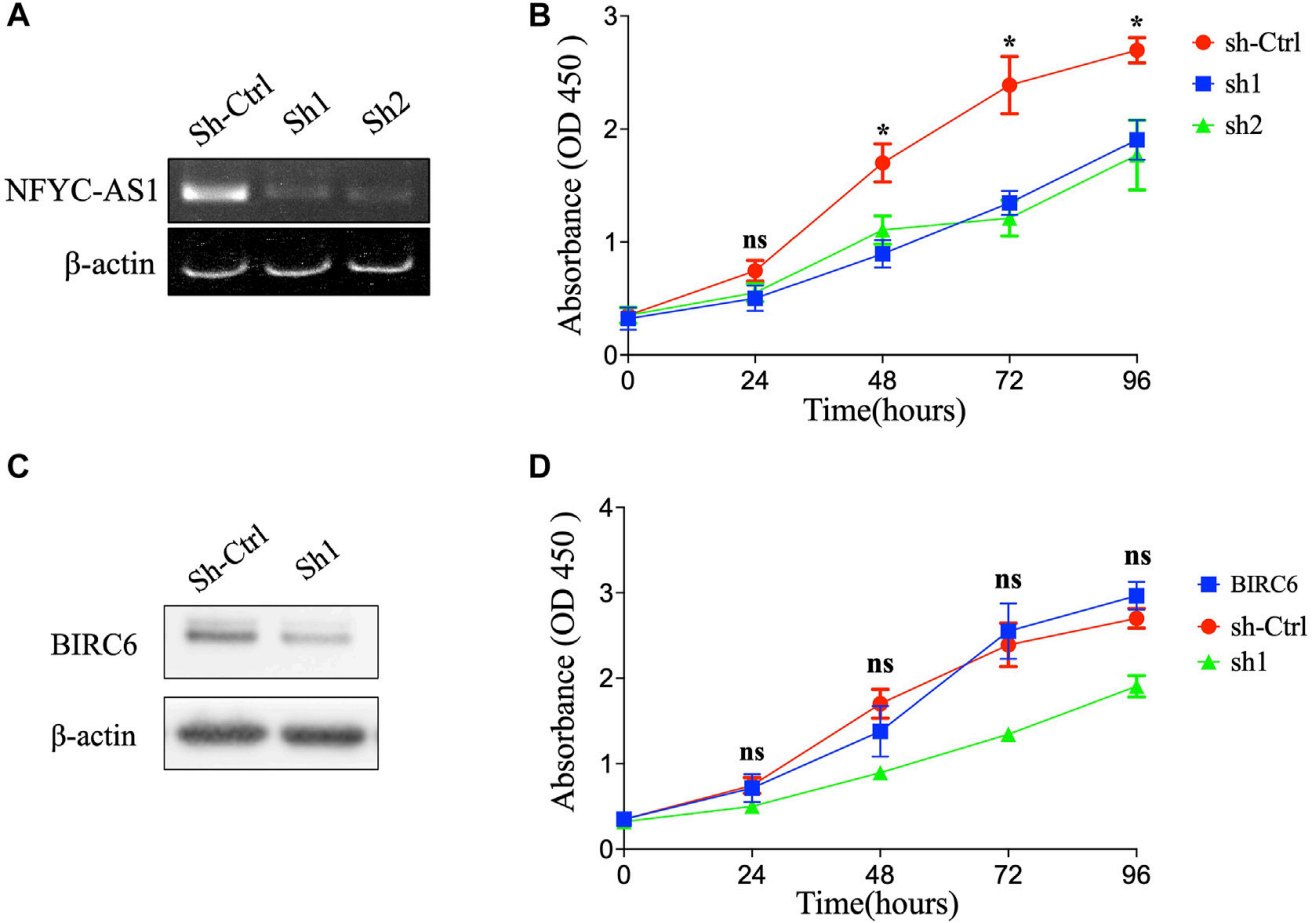
The influence of NFYC-AS1 and BIRC6 on the proliferation of LUAD cell lines. **(A)** RT-PCR results of NFYC-AS1 expression in A549 cells with or without shRNA knockdown. **(B)** The cell proliferation of A549 cells with or without knockdown of NFYC-AS1 expression. **(C)** Immunoblot of BIRC6 expression in A549 cells with or without knockdown of NFYC-AS1 expression. **(D)** The cell proliferation of A549 cells with knockdown of NFYC-AS1 expression or the rescue of BIRC6 expression.

**FIGURE 12:**
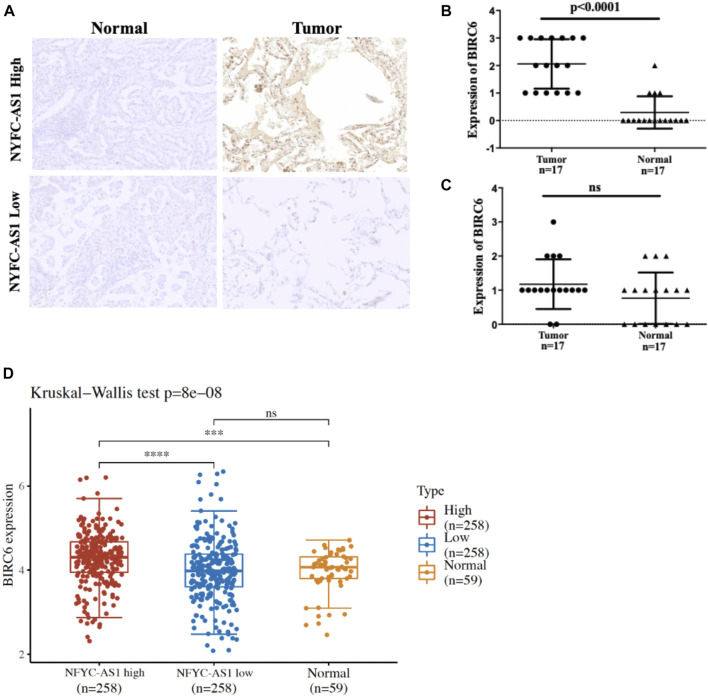
Immunohistochemistry results of 34 pair of tissues from LUAD patients. **(A)** Representative results of BIRC6 expression in normal and tumor tissues based on the median of NFYC-AS1 expression level. **(B)** The quantitative scores of above IHC results based on the higher level of NFYC-AS1 expression. **(C)** The quantitative scores of above IHC results based on the lower level of NFYC-AS1 expression. **(D)** BIRC6 expressions from TCGA database based on the median of NFYC-AS1 expression level.

To investigate the possible connection and its influence between lncRNAs and related autophagy genes, we selected two potential players, NFYC-AS1 and BIRC6, based on previous literature (Song et al., 2021) and their high degree of correlation (Figure 5; Supplementary Figure S3A). Correlation analysis from GEPIA confirmed a positive correlation between the NFYC-AS1 and BIRC6 expression in TCGA LUAD dataset (*p* < 0.001, R = 0.35) but a relatively weak correlation in GTEx lung dataset (*p* < 0.05, R = 0.15) (Supplementary Figures S3B,C). Immunoblot results from lung adenocarcinoma cell line showed that knockdown of NFYC-AS1 did reduce the BIRC6 protein level (Figures 11A,C), suggesting a possible regulation of BIRC6 expression by NFYC-AS1. To determine the subsequent effect of this regulation, we explored the influence of NFYC-AS1 and BIRC6 on A549 cells. Results show that the knockdown of NFYC-AS1 clearly inhibits cell growth (Figure 11B), whereas the restoration of BIRC6 expression reverses the inhibitory effect of NFYC-AS1 knockdown (Figure 11D), suggesting that the pro-proliferative effect of NFYC-AS1 is *via* the regulation of BIRC6. More importantly, IHC of BIRC6 from 34 LUAD patients showed significantly lower expression in NFYC-AS1 low or normal lung tissues and a markedly elevated expression in tumor tissues with high NFYC-AS1 expression (Figures 12A,B,C). In addition, mRNA expression pattern of BIRC6 from the TCGA database was consistent with the above results from patient samples (Figure 12D), both indicating that NFYC-AS1 may promote tumor progress and act as a high-risk factor of poor prognosis in our constructed signature through the upregulation of BIRC6.

**FIGURE 13:**
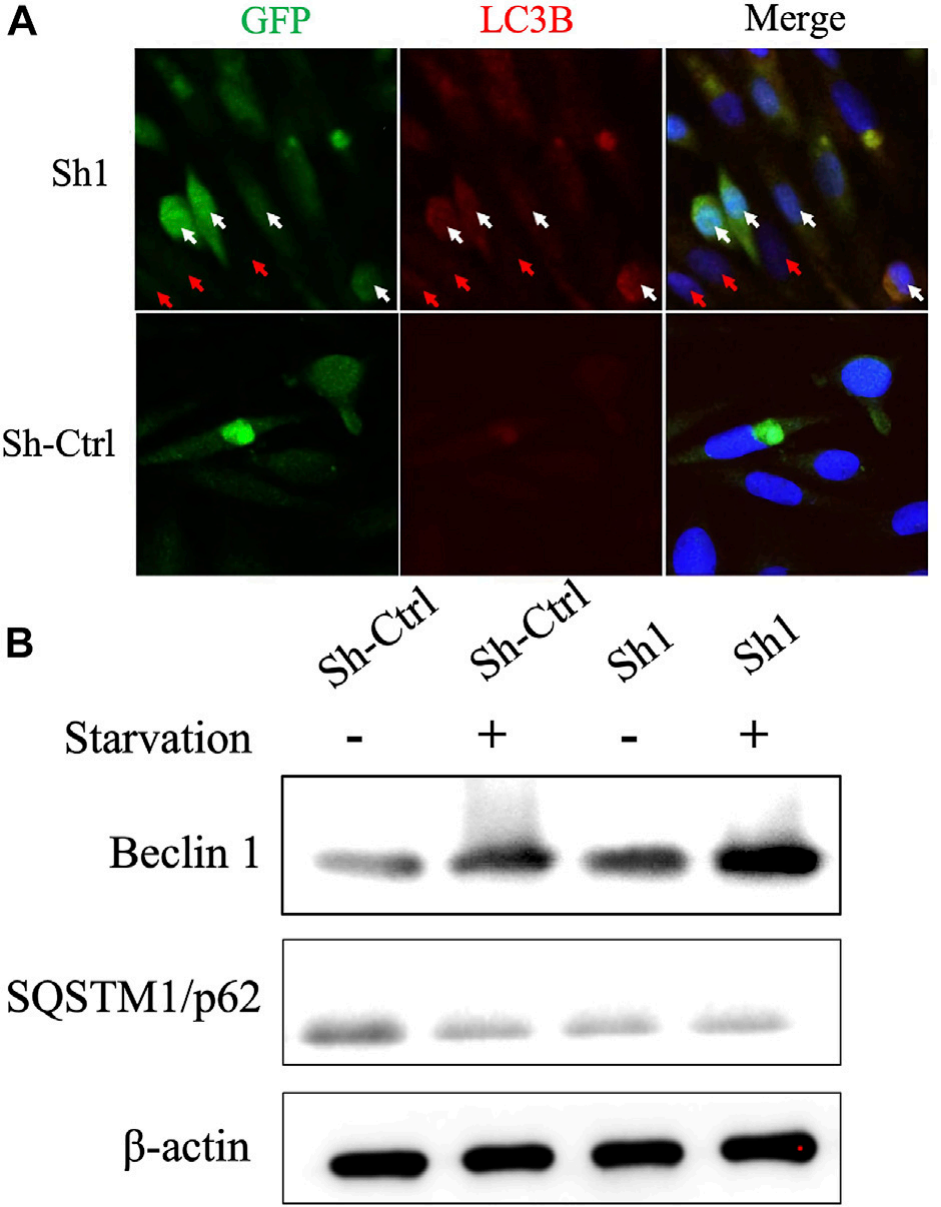
The influence of NFYC-AS1 on the autophagy of A549 cells. **(A)** Immunofluorescence analysis of key autophagy marker LC3B in A549 cells with or without knockdown of NFYC-AS1 expression. The white arrow indicates the significant expression of shRNA plasmids, whereas the red arrow indicates a less significant expression. **(B)** Immunoblot of Beclin 1 and SQSTM1/p62 expression in A549 cells with or without knockdown of NFYC-AS1 expression.

**FIGURE 14:**
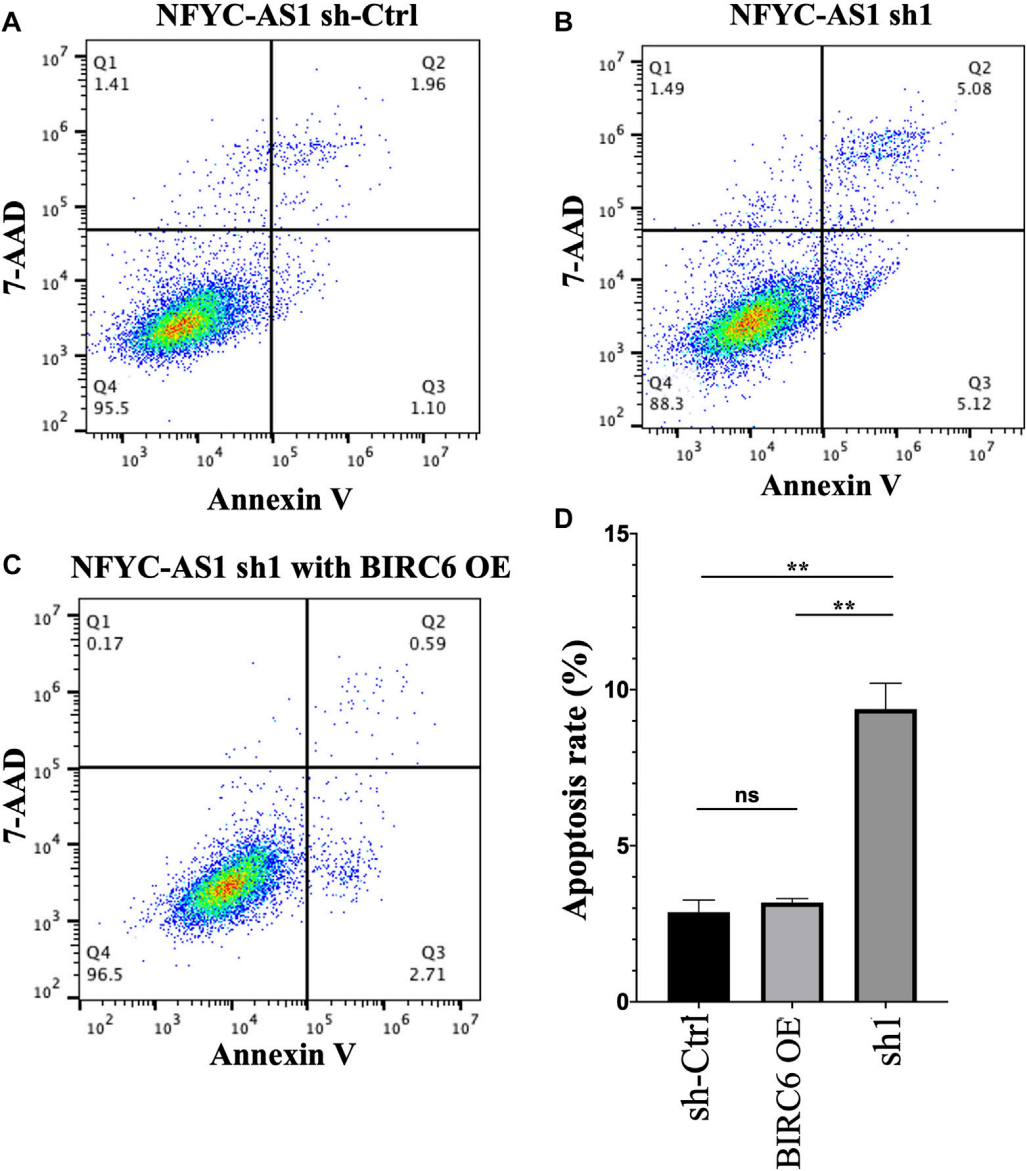
The influence of NFYC-AS1 on the apoptosis of A549 cells. **(A)** Representative flow cytometry analysis of apoptosis in A549 cells without the knockdown of NFYC-AS1 expression. **(B)** Representative flow cytometry analysis of apoptosis in A549 cells with the knockdown of NFYC-AS1 expression. **(C)** Representative flow cytometry analysis of apoptosis in A549 cells with the knockdown of NFYC-AS1 expression combined with the rescue of BIRC6 expression. **(D)** Statistic results of apoptosis rate in A549 cells with or without the knockdown of NFYC-AS1 expression or combining the NFYC-AS1 knockdown with BIRC6 overexpression.

Given that BIRC6 is known to be the regulator of both autophagy and apoptosis (Ebner et al., 2018), we investigated the influence of this regulation on cell autophagy and apoptosis. Results reveal that the knockdown of NFYC-AS1 indeed alters the expression of some autophagy-related markers, including LC3B, Beclin 1, and SQSTM1/p62 in A549 cells (Figures 13A,B). Furthermore, the knockdown of NFYC-AS1 also induced evident apoptosis in A549 cells (Figures 14A,B), whereas BIRC6 overexpression seemingly offset this effect (Figures 14C,D). More importantly, we performed *in vivo* tumor xenograft experiment in nude mice and found that the knockdown of NFYC-AS1 did exhibit the inhibitory effect on tumor growth, whereas the overexpression of BIRC6 could restore the tumor growth in nude mice (Supplementary Figure S5).

## Discussion

As the most common pathological subtype of lung cancer, the proportion of lung adenocarcinoma (LUAD) is up to 40%–70% of all lung cancer patients. Although certain progress has been made in the research and practice of LUAD treatment, it is still one of the most difficult types of cancers to treat due to its highly metastatic and malignant nature. The dysregulation of autophagy is thought to play an important role in lung cancer. For example, in a mouse model of lung cancer, upregulation of autophagy *via* caspase-3 and mTOR inhibition promotes the efficacy of radiotherapy. C-myc/miR-150/EPG5 axis mediated dysfunction of autophagy induced increased cellular ROS levels and DNA damage response and promoted NSCLC development (Li et al., 2019a). Also, autophagy could protect LUAD cells to Src inhibitors, whereas microRNA-106a could target autophagy kinase ULK1 and compromise this protective effect (Rothschild et al., 2017). In addition, autophagy inhibition of cancer stem cells could promote the efficacy of cisplatin against NSCLC in both A549 cell lines and NOD/SCID mice (Hao et al., 2019).

LncRNA, along with miRNA and mRNA expression, has been used to develop risk signatures to prevent and predict the development of various tumors. Increasing evidence has shown significant correlations between lncRNAs and autophagy in lung cancer. For instance, the lncRNA BLACAT1 promoted ATG7 expression through miR-17, facilitated autophagy, and promoted the chemoresistance of NSCLC cells through the miR-17/ATG7 axis (Huang et al., 2019). lncRNA CASC2 inhibited autophagy and promoted apoptosis in NSCLC cells *via* the miR-214/TRIM16 signaling pathway (Li et al., 2018a). LncRNA MSTO2P promotes lung cancer cell proliferation and autophagy by upregulating EZH2 (Wang et al., 2019). Moreover, lncRNA LCPAT1 mediates smoking or PM 2.5-induced autophagy and epithelial–mesenchymal transition *via* RCC2, implying the possible role of lncRNA in rendering lung cancer cells into a more invasive state (Lin et al., 2018).

In this study, we identified and validated an autophagy-related 12-lncRNA risk signature that was highly associated with the OS of patients with LUAD. The signature showed accuracy and robustness by calculating the AUC in the ROC analysis. Multivariate Cox regression analyses suggested that age, N stage, and the risk signature were independent risk factors for OS in the primary cohort. A novel nomogram incorporating clinical factors was constructed and validated to predict the prognosis for LUAD patients. The expression levels of lncRNA signatures were also validated in the clinical LUAD samples by qRT-PCR. These results suggested that both the 12-lncRNA risk signature and the novel nomogram were effective prognostic indicators in patients with LUAD.

SNHG10 has been identified as oncogenic in bladder cancer (Jiang et al., 2018), hepatocellular carcinoma (Lan et al., 2019), osteosarcoma (He et al., 2020), glioma (Jin et al., 2020), non–small cell lung cancer (Zhang et al., 2020b; Liang et al., 2020), acute myeloid leukemia (Xiao et al., 2021), and gastric cancer (Yuan et al., 2021; Zhang et al., 2021). HCG18 was found to be involved in several tumors (Xi et al., 2017; Li et al., 2018b; Wang et al., 2018; Li et al., 2019b; Li et al., 2020a; Liu et al., 2020; Ma et al., 2020; Zhang and Lou, 2020; Zou et al., 2020; Yang et al., 2021; Zhu et al., 2021) and recognized as the driver of LUAD (Li et al., 2020b). It is worth mentioning that although HCG18 is linked to a better prognosis in our risk signature, its expression is higher in tumors compared to adjacent normal tissues, which may be due to the compensatory effect of this lncRNA as a potential tumor suppressor. It has been shown that LINC00857 enhances BIRC5-dependent radio-sensitivity of cancer cells (Han et al., 2020), regulates apoptosis and glycolysis (Wang et al., 2020b), and is identified as one of the prognostic markers for the early stage lung adenocarcinoma (Mu et al., 2021). LINC01116 is oncogenic in several other kinds of tumors (Hu et al., 2018; Chen et al., 2020; Wu et al., 2020; Ye et al., 2020) and promotes lung adenocarcinoma proliferation and metastasis possibly *via* the Akt pathway (Zeng et al., 2020; Shang et al., 2021; Wu et al., 2021). The prognostic function of LINC00996 is also implicated in colorectal cancer (Ge et al., 2018), head and neck cancer (Ge et al., 2018), and multiple myeloma (Zhou et al., 2020). Overexpressed CRNDE was observed in colorectal cancer (Yu et al., 2017), glioma (Wang et al., 2015), hepatocellular carcinoma (Ji et al., 2019), breast cancer (Huan et al., 2017), and gastric cancer (Hu et al., 2017) and was found to be related to the metastasis and radiosensitivity in lung cancer (Zhang et al., 2018; Jing et al., 2019). CASC15 is another unfavorable prognostic marker found in multiple tumors, including lung cancer (Lessard et al., 2015; Fernando et al., 2017; He et al., 2017; Yao et al., 2017; Wu et al., 2018; Bai et al., 2019; Li et al., 2019c; Xie and Cheng, 2019; Yu et al., 2019; Liang et al., 2021; Yu et al., 2021). Previous studies have found that TMPO-AS1 is upregulated and correlated with poor prognosis in LUAD patients (Qin et al., 2019). Bioinformatics analysis also revealed that TMPO-AS1 could affect the prognosis of LUAD by regulating cell cycle pathway genes such as CDC25A (Peng et al., 2017).

Compared to other members in the risk signature, NFYC-AS1 received relatively little attention, except that it has been demonstrated as a prognostic biomarker in the miRNA-lncRNA-mRNA regulatory network in LUAD patients (Li et al., 2016). BIRC6 (Baculoviral IAP repeat-containing 6) is an IAP (inhibitor of apoptosis proteins) that not only plays an anti-apoptotic role through the inhibition of pro-apoptotic protein SMAC or caspases (Hauser et al., 1998; Hao et al., 2004; Qiu and Goldberg, 2005; Ren et al., 2005) but also is associated with other cellular processes; for instance, it regulates autophagosome-lysosome fusion in autophagy (Ikeda, 2018; Jia and Bonifacino, 2019). In addition, overexpression of BIRC6 has been found in various tumors, including colorectal cancer (Bianchini et al., 2006), gastric carcinomas (Salehi et al., 2016), and lung cancer (Dong et al., 2013). In our study, the NFYC-AS1 expression shows a high positive correlation with autophagy genes, especially with BIRC6 (*p* < 0.001, R = 0.35), and the knockdown of NFYC-AS1 decreased the BIRC6 expression and induces both autophagy and apoptosis in A549 cells, whereas the rescue of BIRC6 reverses the inhibitory effect (Figures 13, 14). Although there is no evidence showing a direct regulation of BIRC6 by NFYC-AS1 until now, combining all the above results, it is possible that NFYC-AS1 enhances BIRC6 expression *via* binding to specific proteins or regulating miRNAs, which in turn leads to the inhibition of autophagy, apoptosis, and the progression of tumors.

Gene set enrichment analysis (GSEA) further revealed that the constructed risk signature may regulate several KEGG pathways to influence the progression of LUAD, including the ‘‘p53 signaling,’’ “proteasome,” “protein export,” and “pentose phosphate” pathways. Tumor suppressor p53 acts as the guardian of the genome, which senses the stress in the cellular environment, initiates DNA repair, or leads to the cell cycle arrest. Recently, growing evidence has revealed that p53 can activate the transcription of autophagy-related genes, whereas autophagy could suppress p53 *via* the regulation of ROS or AMPK. In contrast, proteasome is considered responsible for proteolysis and organelle homeostasis by interconnecting with autophagy pathways. As a crucial part of autophagy, lysosomes carry out the degradation of extracellular particles from endocytosis and of intracellular components from autophagy, which contains more than 50 membrane proteins that control the specificity and timing of cargo influx and protein export. The pentose phosphate pathway (PPP) plays a key role in facilitating tumor cells with the glycolytic process and countering the damage of reactive oxidative species. G6PD is the limiting enzyme of the PPP associated with cancer progression and drug resistance, the inhibition of which indirectly leads to the dysfunction of autophagy. Although GESA analysis implicated the involvement of lncRNAs and autophagy in LUAD tumor progression, the detailed association of how autophagy-related tumor progression and lncRNAs are included in our signatures should be further investigated.

This study has some obvious advantages. First, it included 551 samples from the LUAD TCGA dataset and 246 samples from GSE31210, providing a sufficient sample size to avoid statistical bias and make a satisfactory result. Indeed, the constructed signature and incorporated nomogram were further validated in both TCGA train and GSE datasets and showed a good specialty and sensitivity. Second, clinical samples further confirmed that the expressions of several lncRNAs are consistent with the results from the database. Then, a novel nomogram incorporating the risk signature and related clinicopathological factors is constructed and provides a helpful prediction for the prognosis of LUAD patients. Third, we further investigated the possible regulation of BIRC6 by NFYC-AS1 and related influences on lung cancer cells, proving that NFYC-AS1 may act not only as an indicator for poor prognosis but also as a potential driver of carcinogenesis through the negative modulation of autophagy and apoptosis. Although, in current experimental settings, it is difficult to distinguish which effect is dominant or whether BIRC6 is directly responsible for this inhibitory effect through autophagy, all the above results suggest the importance of NFYC-AS1 as a pro-cancer factor in LUAD patients.

In the meantime, there are some limitations to the current study. First, datasets from GEO or TCGA have various kinds of lncRNAs due to their unique sequencing methods. Thus, on the one hand, using both GEO and TCGA datasets may increase the sample size and help the validation of risk signatures. On the other hand, the overlap of two different datasets may neglect some lncRNAs that are significantly deregulated in one database but not sequenced at all in another. Second, the TCGA and GSE31210 databases did not cover important information such as medical history, treatment strategy, and family history, which could alter the prognosis of LUAD patients. Third, although our results showed a possible regulation of NFYC-AS1 on BIRC6 and BIRC6 expression indeed increased in tumor tissues (Supplementary Figure S4), the direct evidence of this regulation still needs to be further discovered. Last, in the current study, we investigated the expression of four lnRNAs in 10 patients’ samples. To assure the accuracy of the expression patterns, clearly more clinical samples should be analyzed.

## Conclusion

Collectively, our current study analyzed autophagy-related lncRNAs in LUAD patients comprehensively and constructed a lncRNA-based risk signature. Our results revealed that high-risk scores are associated with a worse prognosis in LUAD patients. Our study also suggested that the 12 autophagy-related lncRNAs have significant value in predicting the prognosis of LUAD patients. Furthermore, NFYC-AS1 and BIRC6 may be potential therapeutic targets due to previous research and their significance in our study. More specific experiments and research based on clinical samples should be carried out to validate the results of our study.

## Data Availability

The original contributions presented in the study are included in the article/Supplementary Material. Further inquiries can be directed to the corresponding author.

## References

[B1] Bai Y. Zhang G. Cheng R. Yang R. Chu H. (2019). CASC15 contributes to proliferation and invasion through regulating miR-766-5p/KLK12 axis in lung cancer. Cell. Cycle 18, 2323–2331. 10.1080/15384101.2019.1646562 31378128 PMC6738530

[B2] Bianchini M. Levy E. Zucchini C. Pinski V. Macagno C. De Sanctis P. (2006). Comparative study of gene expression by cDNA microarray in human colorectal cancer tissues and normal mucosa. Int. J. Oncol. 29, 83–94. 10.3892/ijo.29.1.83 16773188

[B3] Chen J. Yuan Z. H. Hou X. H. Shi M. H. Jiang R. (2020). LINC01116 promotes the proliferation and inhibits the apoptosis of gastric cancer cells. Eur. Rev. Med. Pharmacol. Sci. 24, 1807–1814. 10.26355/eurrev_202002_20358 32141549

[B4] Dong X. Lin D. Low C. Vucic E. A. English J. C. Yee J. (2013). Elevated expression of BIRC6 protein in non-small-cell lung cancers is associated with cancer recurrence and chemoresistance. J. Thorac. Oncol. 8, 161–170. 10.1097/JTO.0b013e31827d5237 23287853

[B5] Ebner P. Poetsch I. Deszcz L. Hoffmann T. Zuber J. Ikeda F. (2018). The IAP family member BRUCE regulates autophagosome-lysosome fusion. Nat. Commun. 9, 599. 10.1038/s41467-018-02823-x 29426817 PMC5807552

[B6] Fernando T. R. Contreras J. R. Zampini M. Rodriguez-Malave N. I. Alberti M. O. Anguiano J. (2017). The lncRNA CASC15 regulates SOX4 expression in RUNX1-rearranged acute leukemia. Mol. Cancer 16, 126. 10.1186/s12943-017-0692-x 28724437 PMC5517805

[B7] Fulda S. Kögel D. (2015). Cell death by autophagy: Emerging molecular mechanisms and implications for cancer therapy. Oncogene 34, 5105–5113. 10.1038/onc.2014.458 25619832

[B8] Ge H. Yan Y. Wu D. Huang Y. Tian F. (2018). Potential role of LINC00996 in colorectal cancer: A study based on data mining and bioinformatics. Onco. Targets. Ther. 11, 4845–4855. 10.2147/ott.s173225 30147336 PMC6098418

[B9] Gewirtz D. A. (2014). The four faces of autophagy: Implications for cancer therapy. Cancer Res. 74, 647–651. 10.1158/0008-5472.can-13-2966 24459182

[B10] Guan H. Zhu T. Wu S. Liu S. Liu B. Wu J. (2019). Long noncoding RNA LINC00673-v4 promotes aggressiveness of lung adenocarcinoma via activating WNT/β-catenin signaling. Proc. Natl. Acad. Sci. U. S. A. 116, 14019–14028. 10.1073/pnas.1900997116 31235588 PMC6628810

[B11] Han F. Yang S. Wang W. Huang X. Huang D. Chen S. (2020). Silencing of lncRNA LINC00857 enhances BIRC5-dependent radio-sensitivity of lung adenocarcinoma cells by recruiting NF-κB1. Mol. Ther. Nucleic Acids 22, 981–993. 10.1016/j.omtn.2020.09.020 33251047 PMC7679245

[B12] Han J. Hou W. Goldstein L. A. Stolz D. B. Watkins S. C. Rabinowich H. (2014). A complex between Atg7 and caspase-9: A novel mechanism of cross-regulation between autophagy and apoptosis. J. Biol. Chem. 289, 6485–6497. 10.1074/jbc.M113.536854 24362031 PMC3945314

[B13] Hao C. Liu G. Tian G. (2019). Autophagy inhibition of cancer stem cells promotes the efficacy of cisplatin against non-small cell lung carcinoma. Ther. Adv. Respir. Dis. 13, 1753466619866097. 10.1177/1753466619866097 31368411 PMC6676261

[B14] Hao Y. Sekine K. Kawabata A. Nakamura H. Ishioka T. Ohata H. (2004). Apollon ubiquitinates SMAC and caspase-9, and has an essential cytoprotection function. Nat. Cell. Biol. 6, 849–860. 10.1038/ncb1159 15300255

[B15] Hauser H-P. Bardroff M. Pyrowolakis G. JentSch S. (1998). A giant ubiquitin-conjugating enzyme related to IAP apoptosis inhibitors. J. Cell. Biol. 141, 1415–1422. 10.1083/jcb.141.6.1415 9628897 PMC2132795

[B16] He P. Xu Y. Wang Z. (2020). LncRNA SNHG10 increases the methylation of miR-218 gene to promote glucose uptake and cell proliferation in osteosarcoma. J. Orthop. Surg. Res. 15, 353. 10.1186/s13018-020-01865-6 32843060 PMC7448318

[B17] He T. Zhang L. Kong Y. Huang Y. Zhang Y. Zhou D. (2017). Long non-coding RNA CASC15 is upregulated in hepatocellular carcinoma and facilitates hepatocarcinogenesis. Int. J. Oncol. 51, 1722–1730. 10.3892/ijo.2017.4175 29075788 PMC5673007

[B18] Hirata H. Hinoda Y. Shahryari V. Deng G. Nakajima K. Tabatabai Z. L. (2015). Long noncoding RNA MALAT1 promotes aggressive renal cell carcinoma through Ezh2 and interacts with miR-205. Cancer Res. 75, 1322–1331. 10.1158/0008-5472.can-14-2931 25600645 PMC5884967

[B19] Hu C. E. Du P. Z. Zhang H. D. Huang G. J. (2017). Long noncoding RNA CRNDE promotes proliferation of gastric cancer cells by targeting miR-145. Cell. Physiol. biochem. 42, 13–21. 10.1159/000477107 28490034

[B20] Hu H. B. Chen Q. Ding S. Q. (2018). LncRNA LINC01116 competes with miR-145 for the regulation of ESR1 expression in breast cancer. Eur. Rev. Med. Pharmacol. Sci. 22, 1987–1993. 10.26355/eurrev_201804_14726 29687853

[B21] Huan J. Xing L. Lin Q. Xui H. Qin X. (2017). Long noncoding RNA CRNDE activates Wnt/β-catenin signaling pathway through acting as a molecular sponge of microRNA-136 in human breast cancer. Am. J. Transl. Res. 9, 1977–1989. 28469804 PMC5411947

[B22] Huang F. X. Chen H. J. Zheng F. X. Gao Z. Y. Sun P. F. Peng Q. (2019). LncRNA BLACAT1 is involved in chemoresistance of non-small cell lung cancer cells by regulating autophagy. Int. J. Oncol. 54, 339–347. 10.3892/ijo.2018.4614 30387831

[B23] Ikeda F. (2018). The anti-apoptotic ubiquitin conjugating enzyme BIRC6/BRUCE regulates autophagosome-lysosome fusion. Autophagy 14, 1283–1284. 10.1080/15548627.2018.1471311 29929453 PMC6103727

[B24] Iyer M. K. Niknafs Y. S. Malik R. Singhal U. Sahu A. Hosono Y. (2015). The landscape of long noncoding RNAs in the human transcriptome. Nat. Genet. 47, 199–208. 10.1038/ng.3192 25599403 PMC4417758

[B25] Ji D. Jiang C. Zhang L. Liang N. Jiang T. Yang B. (2019). LncRNA CRNDE promotes hepatocellular carcinoma cell proliferation, invasion, and migration through regulating miR-203/BCAT1 axis. J. Cell. Physiol. 234, 6548–6560. 10.1002/jcp.27396 30230527

[B26] Jia R. Bonifacino J. S. (2019). Negative regulation of autophagy by UBA6-BIRC6–mediated ubiquitination of LC3. Elife 8, e50034. 10.7554/eLife.50034 31692446 PMC6863627

[B27] Jiang B. Hailong S. Yuan J. Zhao H. Xia W. Zha Z. (2018). Identification of oncogenic long noncoding RNA SNHG12 and DUXAP8 in human bladder cancer through a comprehensive profiling analysis. Biomed. Pharmacother. = Biomedecine Pharmacother. 108, 500–507. 10.1016/j.biopha.2018.09.025 30243082

[B28] Jin L. Huang S. Guan C. Chang S. (2020). ETS1-activated SNHG10 exerts oncogenic functions in glioma via targeting miR-532-3p/FBXL19 axis. Cancer Cell. Int. 20, 589. 10.1186/s12935-020-01649-2 33298070 PMC7725120

[B29] Jing H. Xia H. Qian M. Lv X. (2019). Long noncoding RNA CRNDE promotes non-small cell lung cancer progression via sponging microRNA-338-3p. Biomed. Pharmacother. = Biomedecine Pharmacother. 110, 825–833. 10.1016/j.biopha.2018.12.024 30554121

[B30] Klionsky D. J. (2007). Autophagy: From phenomenology to molecular understanding in less than a decade. Nat. Rev. Mol. Cell. Biol. 8, 931–937. 10.1038/nrm2245 17712358

[B31] Lan T. Yuan K. Yan X. Xu L. Liao H. Hao X. (2019). LncRNA SNHG10 facilitates hepatocarcinogenesis and metastasis by modulating its homolog SCARNA13 via a positive feedback loop. Cancer Res. 79, 3220–3234. 10.1158/0008-5472.can-18-4044 31101763

[B32] Lessard L. Liu M. Marzese D. M. Wang H. Chong K. Kawas N. (2015). The CASC15 long intergenic noncoding RNA locus is involved in melanoma progression and phenotype switching. J. Invest. Dermatol. 135, 2464–2474. 10.1038/jid.2015.200 26016895 PMC4567947

[B33] Li D. S. Ainiwaer J. L. Sheyhiding I. Zhang Z. Zhang L. W. (2016). Identification of key long non-coding RNAs as competing endogenous RNAs for miRNA-mRNA in lung adenocarcinoma. Eur. Rev. Med. Pharmacol. Sci. 20, 2285–2295. 27338053

[B34] Li H. Liu J. Cao W. Xiao X. Liang L. Liu-Smith F. (2019). C-myc/miR-150/EPG5 axis mediated dysfunction of autophagy promotes development of non-small cell lung cancer. Theranostics 9, 5134–5148. 10.7150/thno.34887 31410206 PMC6691579

[B35] Li L. Ma T. T. Ma Y. H. Jiang Y. F. (2019). LncRNA HCG18 contributes to nasopharyngeal carcinoma development by modulating miR-140/CCND1 and Hedgehog signaling pathway. Eur. Rev. Med. Pharmacol. Sci. 23, 10387–10399. 10.26355/eurrev_201912_19678 31841193

[B36] Li M. Chen Y. Zhu J. Gao Z. Wang T. Zhou P. (2019). Long noncoding RNA CASC15 predicts unfavorable prognosis and exerts oncogenic functions in non-small cell lung cancer. Am. J. Transl. Res. 11, 4303–4314. 31396336 PMC6684884

[B37] Li M. Izpisua Belmonte J. C. (2015). Roles for noncoding RNAs in cell-fate determination and regeneration. Nat. Struct. Mol. Biol. 22, 2–4. 10.1038/nsmb.2946 25565025

[B38] Li Q. Chen K. Dong R. Lu H. (2018). LncRNA CASC2 inhibits autophagy and promotes apoptosis in non-small cell lung cancer cells via regulating the miR-214/TRIM16 axis. RSC Adv. 8, 40846–40855. 10.1039/c8ra09573f 35557905 PMC9091572

[B39] Li S. Wu T. Zhang D. Sun X. Zhang X. (2020). The long non-coding RNA HCG18 promotes the growth and invasion of colorectal cancer cells through sponging miR-1271 and upregulating MTDH/Wnt/β-catenin. Clin. Exp. Pharmacol. Physiol. 47, 703–712. 10.1111/1440-1681.13230 31854468

[B40] Li W. Pan T. Jiang W. Zhao H. (2020). HCG18/miR-34a-5p/HMMR axis accelerates the progression of lung adenocarcinoma. Biomed. Pharmacother. = Biomedecine Pharmacother. 129, 110217. 10.1016/j.biopha.2020.110217 32559619

[B41] Li Y. J. Lei Y. H. Yao N. Wang C. R. Hu N. Ye W. C. (2017). Autophagy and multidrug resistance in cancer. Chin. J. Cancer 36, 52. 10.1186/s40880-017-0219-2 28646911 PMC5482965

[B42] Li Z. Li X. Chen C. Li S. Shen J. Tse G. (2018). Long non-coding RNAs in nucleus pulposus cell function and intervertebral disc degeneration. Cell. Prolif. 51, e12483. 10.1111/cpr.12483 30039593 PMC6528936

[B43] Liang C. Wang J. Liu A. Wu Y. (2021). Tumor promoting long non-coding RNA CASC15 affects HMGB2 expression by sponging miR-582-5p in colorectal cancer. J. Gene Med. 24, e3308. 10.1002/jgm.3308 33395735

[B44] Liang M. Wang L. Cao C. Song S. Wu F. (2020). LncRNA SNHG10 is downregulated in non-small cell lung cancer and predicts poor survival. BMC Pulm. Med. 20, 273. 10.1186/s12890-020-01281-w 33081752 PMC7574240

[B45] Lin H. Zhang X. Feng N. Wang R. Zhang W. Deng X. (2018). LncRNA LCPAT1 mediates smoking/particulate matter 2.5-induced cell autophagy and epithelial-mesenchymal transition in lung cancer cells via RCC2. Cell. Physiol. biochem. 47, 1244–1258. 10.1159/000490220 29913439

[B46] Liu G. Pei F. Yang F. Li L. Amin A. D. Liu S. (2017). Role of autophagy and apoptosis in non-small-cell lung cancer. Int. J. Mol. Sci. 18 (2), 367. 10.3390/ijms18020367 PMC5343902 28208579

[B47] Liu Y. Lin W. Dong Y. Li X. Lin Z. Jia J. (2020). Long noncoding RNA HCG18 up-regulates the expression of WIPF1 and YAP/TAZ by inhibiting miR-141-3p in gastric cancer. Cancer Med. 9, 6752–6765. 10.1002/cam4.3288 32725768 PMC7520348

[B48] Ma F. An K. Li Y. (2020). Silencing of long non-coding RNA-HCG18 inhibits the tumorigenesis of gastric cancer through blocking PI3K/Akt pathway. Onco. Targets. Ther. 13, 2225–2234. 10.2147/ott.s240965 32256081 PMC7092690

[B49] Mu L. Ding K. Tu R. Yang W. (2021). Identification of 4 immune cells and a 5-lncRNA risk signature with prognosis for early-stage lung adenocarcinoma. J. Transl. Med. 19, 127. 10.1186/s12967-021-02800-x 33771173 PMC8004399

[B50] Peng F. Wang R. Zhang Y. Zhao Z. Zhou W. Chang Z. (2017). Differential expression analysis at the individual level reveals a lncRNA prognostic signature for lung adenocarcinoma. Mol. Cancer 16, 98. 10.1186/s12943-017-0666-z 28587642 PMC5461634

[B51] Peng Z. Wang J. Shan B. Li B. Peng W. Dong Y. (2018). The long noncoding RNA LINC00312 induces lung adenocarcinoma migration and vasculogenic mimicry through directly binding YBX1. Mol. Cancer 17, 167. 10.1186/s12943-018-0920-z 30470227 PMC6260658

[B52] Qin Z. Zheng X. Fang Y. (2019). Long noncoding RNA TMPO-AS1 promotes progression of non-small cell lung cancer through regulating its natural antisense transcript TMPO. Biochem. Biophys. Res. Commun. 516, 486–493. 10.1016/j.bbrc.2019.06.088 31230752

[B53] Qiu X-B. Goldberg A. L. (2005). The membrane-associated inhibitor of apoptosis protein, BRUCE/Apollon, antagonizes both the precursor and mature forms of Smac and caspase-9. J. Biol. Chem. 280, 174–182. 10.1074/jbc.M411430200 15507451

[B54] Ren J. Shi M. Liu R. Yang Q. H. Johnson T. Skarnes W. C. (2005). The Birc6 (Bruce) gene regulates p53 and the mitochondrial pathway of apoptosis and is essential for mouse embryonic development. Proc. Natl. Acad. Sci. U. S. A. 102, 565–570. 10.1073/pnas.0408744102 15640352 PMC543482

[B55] Rothschild S. I. Gautschi O. Batliner J. Gugger M. Fey M. F. Tschan M. P. (2017). MicroRNA-106a targets autophagy and enhances sensitivity of lung cancer cells to Src inhibitors. Lung Cancer 107, 73–83. 10.1016/j.lungcan.2016.06.004 27372519

[B56] Salehi S. Jafarian A. H. Montazer M. Moghbeli M. Forghanifard M. M. (2016). BRUCE protein, new marker for targeted therapy of gastric carcinoma. J. Gastrointest. Cancer 48, 151–155. 10.1007/s12029-016-9874-9 27614745

[B57] Shang B. Li Z. Li M. Jiang S. Feng Z. Cao Z. (2021). Silencing LINC01116 suppresses the development of lung adenocarcinoma via the AKT signaling pathway. Thorac. Cancer 12, 2093–2103. 10.1111/1759-7714.14042 34061456 PMC8287011

[B58] Song Y. Du J. Lu P. Zou Q. Zeng S. Liu M. (2021). LncRNA NFYC-AS1 promotes the development of lung adenocarcinomas through autophagy, apoptosis, and MET/c-Myc oncogenic proteins. Ann. Transl. Med. 9, 1621. 10.21037/atm-21-4995 34926665 PMC8640918

[B59] Tamang S. Acharya V. Roy D. Sharma R. Aryaa A. Sharma U. (2019). SNHG12: An LncRNA as a potential therapeutic target and biomarker for human cancer. Front. Oncol. 9, 901. 10.3389/fonc.2019.00901 31620362 PMC6759952

[B60] Ulitsky I. Bartel D. P. (2013). lincRNAs: genomics, evolution, and mechanisms. Cell. 154, 26–46. 10.1016/j.cell.2013.06.020 23827673 PMC3924787

[B61] Wang J. Gao J. Chen Q. Zou W. Yang F. Wei C. (2020). LncRNA LINC01116 contributes to cisplatin resistance in lung adenocarcinoma. Onco. Targets. Ther. 13, 9333–9348. 10.2147/ott.s244879 33061421 PMC7519870

[B62] Wang L. Cao L. Wen C. Li J. Yu G. Liu C. (2020). LncRNA LINC00857 regulates lung adenocarcinoma progression, apoptosis and glycolysis by targeting miR-1179/SPAG5 axis. Hum. Cell. 33, 195–204. 10.1007/s13577-019-00296-8 31667785

[B63] Wang L. J. Sun G. Z. Chen Y. F. (2019). LncRNA MSTO2P promotes proliferation and autophagy of lung cancer cells by up-regulating EZH2 expression. Eur. Rev. Med. Pharmacol. Sci. 23, 3375–3382. 10.26355/eurrev_201904_17701 31081092

[B64] Wang W. Zhao Z. Yang F. Wang H. Wu F. Liang T. (2018). An immune-related lncRNA signature for patients with anaplastic gliomas. J. Neurooncol. 136, 263–271. 10.1007/s11060-017-2667-6 29170907

[B65] Wang Y. Wang Y. Li J. Zhang Y. Yin H. Han B. (2015). CRNDE, a long-noncoding RNA, promotes glioma cell growth and invasion through mTOR signaling. Cancer Lett. 367, 122–128. 10.1016/j.canlet.2015.03.027 25813405

[B66] Wu J. Chen Z. Zhang L. Cao J. Li X. Gong Z. (2020). Knockdown of LINC01116 inhibits cell migration and invasion in head and neck squamous cell carcinoma through epithelial-mesenchymal transition pathway. J. Cell. Biochem. 121, 867–875. 10.1002/jcb.29331 31452270

[B67] Wu L. Wen Z. Song Y. Wang L. (2021). A novel autophagy-related lncRNA survival model for lung adenocarcinoma. J. Cell. Mol. Med. 25, 5681–5690. 10.1111/jcmm.16582 33987935 PMC8184679

[B68] Wu Q. Xiang S. Ma J. Hui P. Wang T. Meng W. (2018). Long non-coding RNA CASC15 regulates gastric cancer cell proliferation, migration and epithelial mesenchymal transition by targeting CDKN1A and ZEB1. Mol. Oncol. 12, 799–813. 10.1002/1878-0261.12187 29489064 PMC5983148

[B69] Xi Y. Jiang T. Wang W. Yu J. Wang Y. Wu X. (2017). Long non-coding HCG18 promotes intervertebral disc degeneration by sponging miR-146a-5p and regulating TRAF6 expression. Sci. Rep. 7, 13234. 10.1038/s41598-017-13364-6 29038477 PMC5643303

[B70] Xiao S. Zha Y. Zhu H. (2021). miR-621 may suppress cell proliferation via targeting lncRNA SNHG10 in acute myeloid leukemia. Cancer Manag. Res. 13, 2117–2123. 10.2147/cmar.s269528 33688254 PMC7936933

[B71] Xie Y. Cheng Y. (2019). Long noncoding RNA CASC15 is upregulated in glioma and facilitates cell proliferation and metastasis via targeting miR-130b-3p. Eur. Rev. Med. Pharmacol. Sci. 23, 7475–7481. 10.26355/eurrev_201909_18857 31539135

[B72] Yang Y. Gong P. Yao D. Xue D. He X. (2021). LncRNA HCG18 promotes clear cell renal cell carcinoma progression by targeting miR-152-3p to upregulate RAB14. Cancer Manag. Res. 13, 2287–2294. 10.2147/cmar.s298649 33732021 PMC7959199

[B73] Yao X. M. Tang J. H. Zhu H. Jing Y. (2017). High expression of LncRNA CASC15 is a risk factor for gastric cancer prognosis and promote the proliferation of gastric cancer. Eur. Rev. Med. Pharmacol. Sci. 21, 5661–5667. 10.26355/eurrev_201712_14010 29272000

[B74] Ye J. Zhu J. Chen H. Qian J. Zhang L. Wan Z. (2020). A novel lncRNA-LINC01116 regulates tumorigenesis of glioma by targeting VEGFA. Int. J. Cancer 146, 248–261. 10.1002/ijc.32483 31144303

[B75] Yin D. Lu X. Su J. He X. De W. Yang J. (2018). Long noncoding RNA AFAP1-AS1 predicts a poor prognosis and regulates non-small cell lung cancer cell proliferation by epigenetically repressing p21 expression. Mol. Cancer 17, 92. 10.1186/s12943-018-0836-7 29793547 PMC5968553

[B76] Yu B. Ye X. Du Q. Zhu B. Zhai Q. Li X. X. (2017). The long non-coding RNA CRNDE promotes colorectal carcinoma progression by competitively binding miR-217 with TCF7L2 and enhancing the wnt/β-catenin signaling pathway. Cell. Physiol. biochem. 41, 2489–2502. 10.1159/000475941 28472810

[B77] Yu D. J. Zhong M. Wang W. L. (2021). Long noncoding RNA CASC15 is upregulated in non-small cell lung cancer and facilitates cell proliferation and metastasis via targeting miR-130b-3p. Eur. Rev. Med. Pharmacol. Sci. 25, 7943–7949. 10.26355/eurrev_201909_19010 31599419

[B78] Yu X. Wang Z. L. Han C. L. Wang M. W. Jin Y. Jin X. B. (2019). LncRNA CASC15 functions as an oncogene by sponging miR-130b-3p in bladder cancer. Eur. Rev. Med. Pharmacol. Sci. 23, 9814–9820. 10.26355/eurrev_201911_19544 31799648

[B79] Yuan S. X. Wang J. Yang F. Tao Q. f. Zhang J. Wang L. l. (2016). Long noncoding RNA DANCR increases stemness features of hepatocellular carcinoma by derepression of CTNNB1. Hepatology 63, 499–511. Article. 10.1002/hep.27893 25964079

[B80] Yuan X. Yang T. Xu Y. Ou S. Shi P. Cao M. (2021). SNHG10 promotes cell proliferation and migration in gastric cancer by targeting miR-495-3p/CTNNB1 Axis. Dig. Dis. Sci. 66, 2627–2636. 10.1007/s10620-020-06576-w 32920660

[B81] Yue B. Qiu S. Zhao S. Liu C. Zhang D. Yu F. (2016). LncRNA-ATB mediated E-cadherin repression promotes the progression of colon cancer and predicts poor prognosis. J. Gastroenterol. Hepatol. 31, 595–603. 10.1111/jgh.13206 26487301

[B82] Zeng L. Lyu X. Yuan J. Wang W. Zhao N. Liu B. (2020). Long non-coding RNA LINC01116 is overexpressed in lung adenocarcinoma and promotes tumor proliferation and metastasis. Am. J. Transl. Res. 12, 4302–4313. 32913506 PMC7476163

[B83] Zhang J. Lou W. (2020). A key mRNA-miRNA-lncRNA competing endogenous RNA triple sub-network linked to diagnosis and prognosis of hepatocellular carcinoma. Front. Oncol. 10, 340. 10.3389/fonc.2020.00340 32257949 PMC7092636

[B84] Zhang M. Gao C. Yang Y. Li G. Dong J. Ai Y. (2018). Long noncoding RNA CRNDE/PRC2 participated in the radiotherapy resistance of human lung adenocarcinoma through targeting p21 expression. Oncol. Res. 26, 1245–1255. 10.3727/096504017x14944585873668 28550688 PMC7844700

[B85] Zhang M. Wei Y. Liu Y. Guan W. Zhang X. Kong J. (2020). Metastatic phosphatase PRL-3 induces ovarian cancer stem cell sub-population through phosphatase-independent deacetylation modulations. iScience 23, 100766. 10.1016/j.isci.2019.100766 31887658 PMC6941878

[B86] Zhang Y. Guo H. Zhang H. (2021). SNHG10/DDX54/PBX3 feedback loop contributes to gastric cancer cell growth. Dig. Dis. Sci. 66, 1875–1884. 10.1007/s10620-020-06488-9 32712782

[B87] Zhang Z. Nong L. Chen M. L. Gu X. L. Zhao W. W. Liu M. H. (2020). Long noncoding RNA SNHG10 sponges miR-543 to upregulate tumor suppressive SIRT1 in nonsmall cell lung cancer. Cancer biother. Radiopharm. 35, 771–775. 10.1089/cbr.2019.3334 32319822

[B88] Zhou S. Fang J. Sun Y. Li H. (2020). Integrated analysis of a risk score system predicting prognosis and a ceRNA network for differentially expressed lncRNAs in multiple myeloma. Front. Genet. 11, 934. 10.3389/fgene.2020.00934 33193574 PMC7481452

[B89] Zhu Y. Zhao J. Tan L. Lin S. Long M. Peng X. (2021). LncRNA-HCG18 regulates the viability, apoptosis, migration, invasion and epithelial-mesenchymal transition of papillary thyroid cancer cells via regulating the miR-106a-5p/PPP2R2A axis. Pathol. Res. Pract. 221, 153395. 10.1016/j.prp.2021.153395 33798913

[B90] Zou Y. Sun Z. Sun S. (2020). LncRNA HCG18 contributes to the progression of hepatocellular carcinoma via miR-214-3p/CENPM axis. J. Biochem. 168, 535–546. 10.1093/jb/mvaa073 32663252

