# Corrigendum: Identification of an autophagy-related 12-lncRNA signature and evaluation of NFYC-AS1 as a pro-cancer factor in lung adenocarcinoma

**DOI:** 10.3389/fgene.2022.1050296

**Published:** 2022-10-25

**Authors:** Fang Tong, Lifa Xu, Sheng Xu, Mingming Zhang

**Affiliations:** ^1^ Department of Medical Immunology, School of Medicine, Anhui University of Science and Technology, Anhui, China; ^2^ Anhui Province Engineering Laboratory of Occupational Health and Safety, Anhui University of Science and Technology, Anhui, China; ^3^ The First Affiliated Hospital, Anhui University of Science and Technology, Anhui, China; ^4^ Chongqing Key Laboratory of Traditional Chinese Medicine for Prevention and Cure of Metabolic Diseases, College of Traditional Chinese Medicine, Chongqing Medical University, Chongqing, China

**Keywords:** LUAD, lncRNA, long noncoding RNA, autophagy, risk signature, prognostic indicator, NFYC-AS1, BIRC6

In the published article, there were errors in the following affiliations:

Instead of “[1] Chongqing Key Laboratory of Traditional Chinese Medicine for Prevention and Cure of Metabolic Diseases, College of Traditional Chinese Medicine, Chongqing Medical University, Chongqing, China”, it should be “[4] Chongqing Key Laboratory of Traditional Chinese Medicine for Prevention and Cure of Metabolic Diseases, College of Traditional Chinese Medicine, Chongqing Medical University, Chongqing, China”.

Instead of “[2] Department of Medical Immunology, School of Medicine, Anhui University of Science and Technology, Chongqing, China”, it should be “[1] Department of Medical Immunology, School of Medicine, Anhui University of Science and Technology, Anhui, China”.

Instead of “[3] Anhui Province Engineering Laboratory of Occupational Health and Safety, Anhui University of Science and Technology, Chongqing, China”, it should be “[2] Anhui Province Engineering Laboratory of Occupational Health and Safety, Anhui University of Science and Technology, Anhui, China”.

Instead of “[4] The First Affiliated Hospital, Anhui University of Science and Technology, Chongqing, China”, it should be “[3] The First Affiliated Hospital, Anhui University of Science and Technology, Anhui, China”.

Also author affiliations have been reordered and affiliation “Chongqing Key Laboratory of Traditional Chinese Medicine for Prevention and Cure of Metabolic Diseases, College of Traditional Chinese Medicine, Chongqing Medical University, Chongqing, China” was removed for author Fang Tong.

The authors apologize for this error and state that this does not change the scientific conclusions of the article in any way. The original article has been updated.

