# Supplementary figures and images for: Identification of an autophagy-related 12-lncRNA signature and evaluation of NFYC-AS1 as a pro-cancer factor in lung adenocarcinoma
Source: Front Genet. 2022 Aug 11;13:834935. doi: 10.3389/fgene.2022.834935 (PMC9466988; doi:10.3389/fgene.2022.834935)

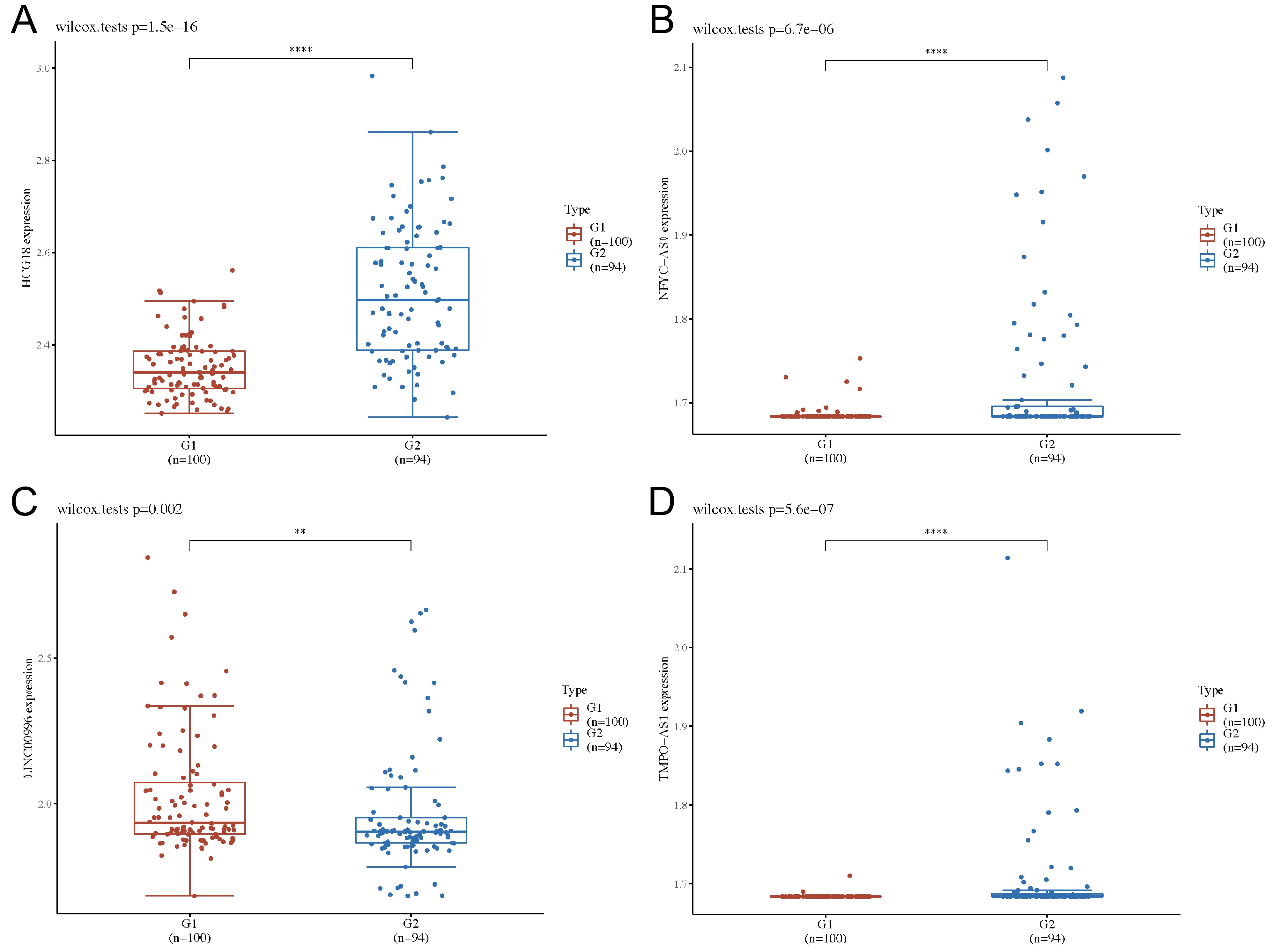

Supplement: Supplementary file 2 [file Image3.JPEG]

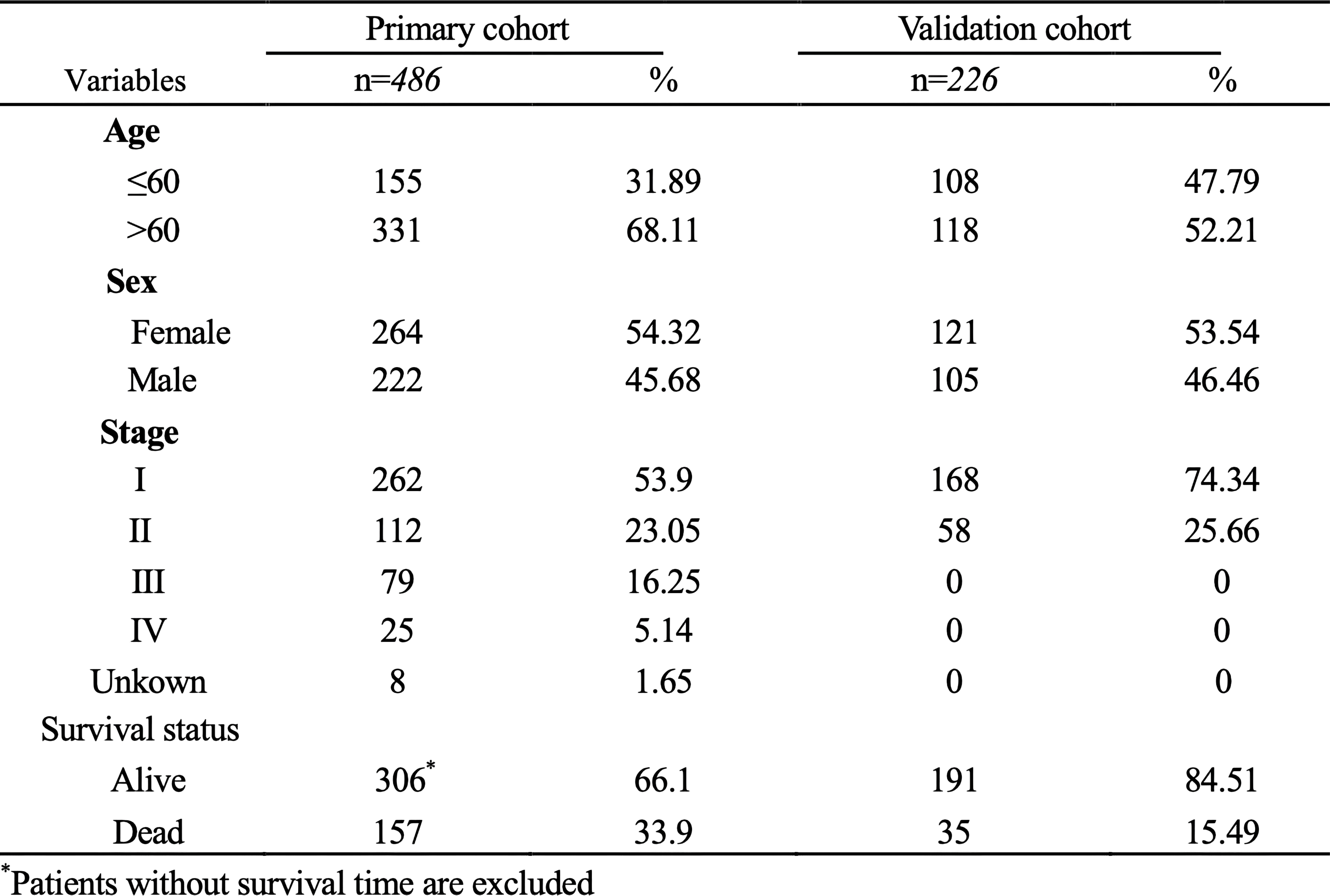

Supplement: Supplementary file 3 [file Image1.JPEG]

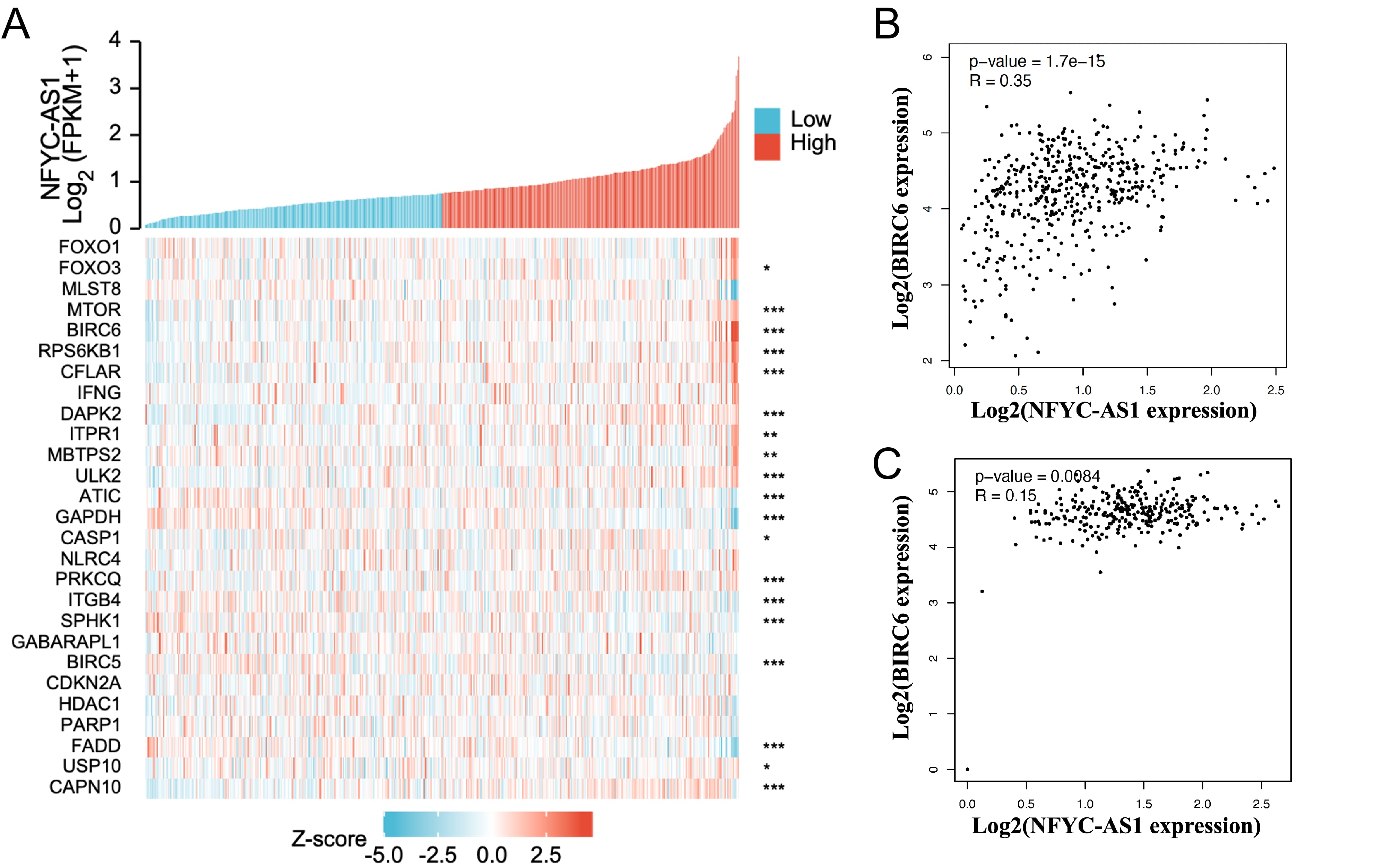

Supplement: Supplementary file 4 [file Image4.JPEG]

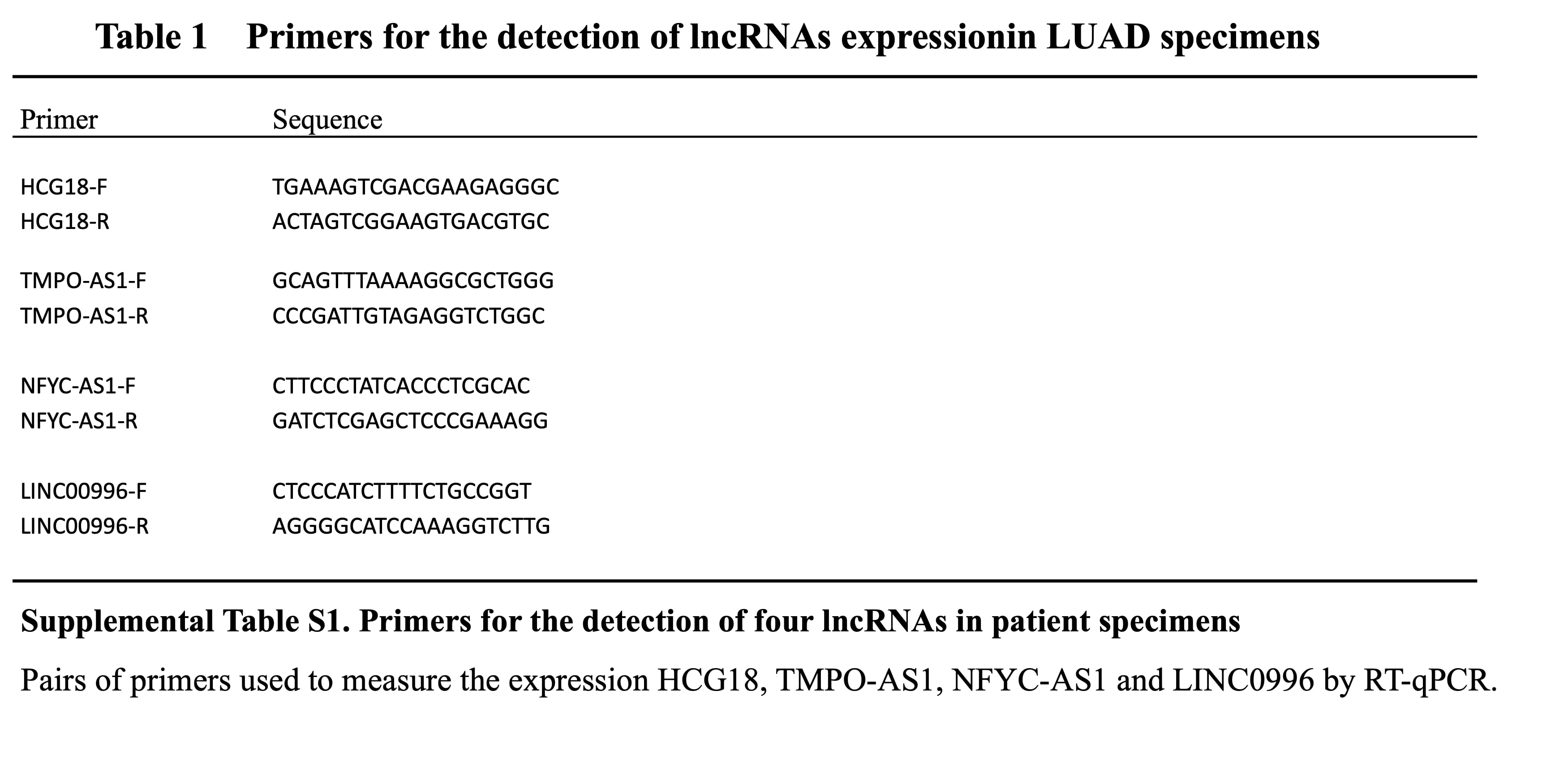

Supplement: Supplementary file 5 [file Image7.JPEG]

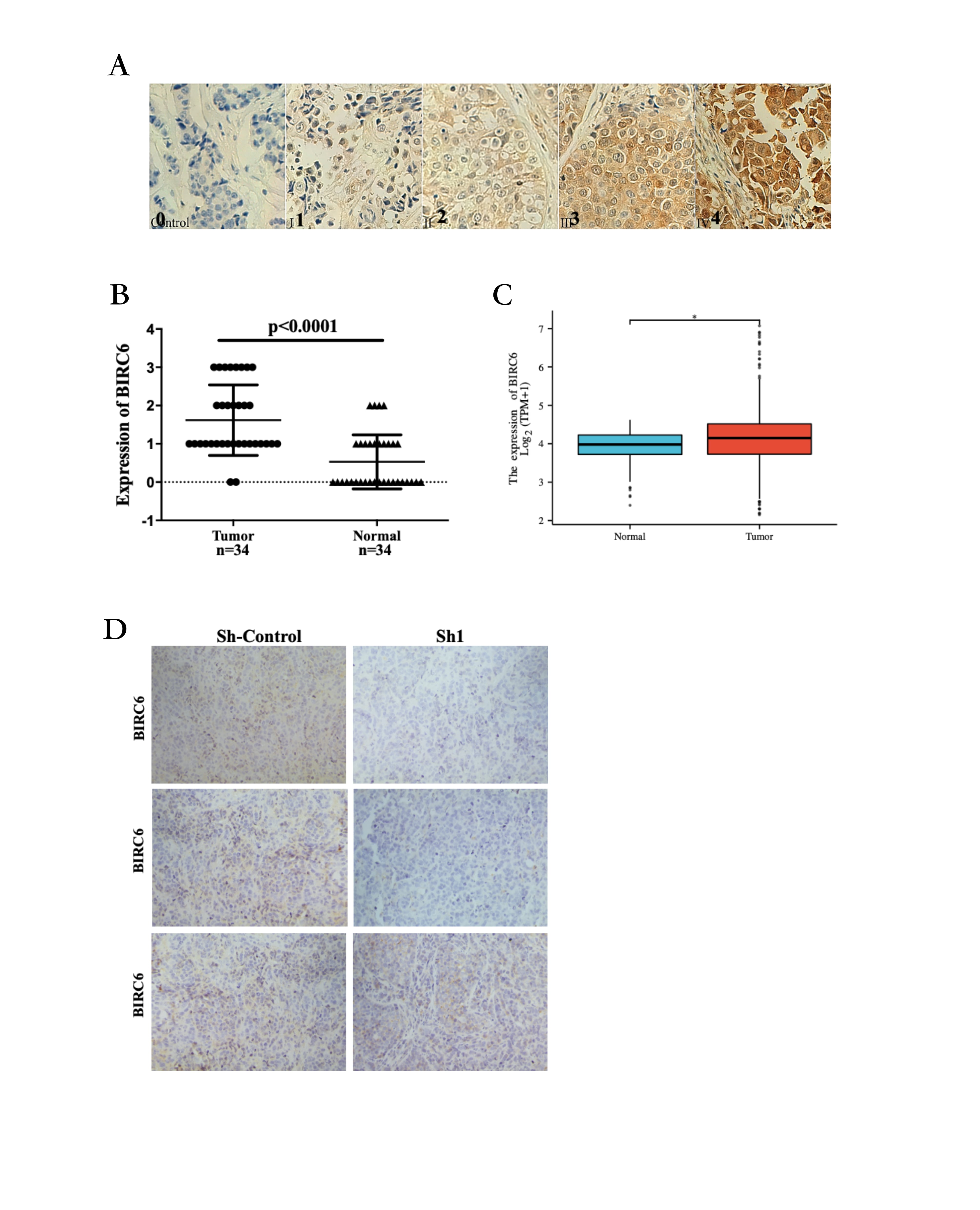

Supplement: Supplementary file 7 [file Image5.JPEG]

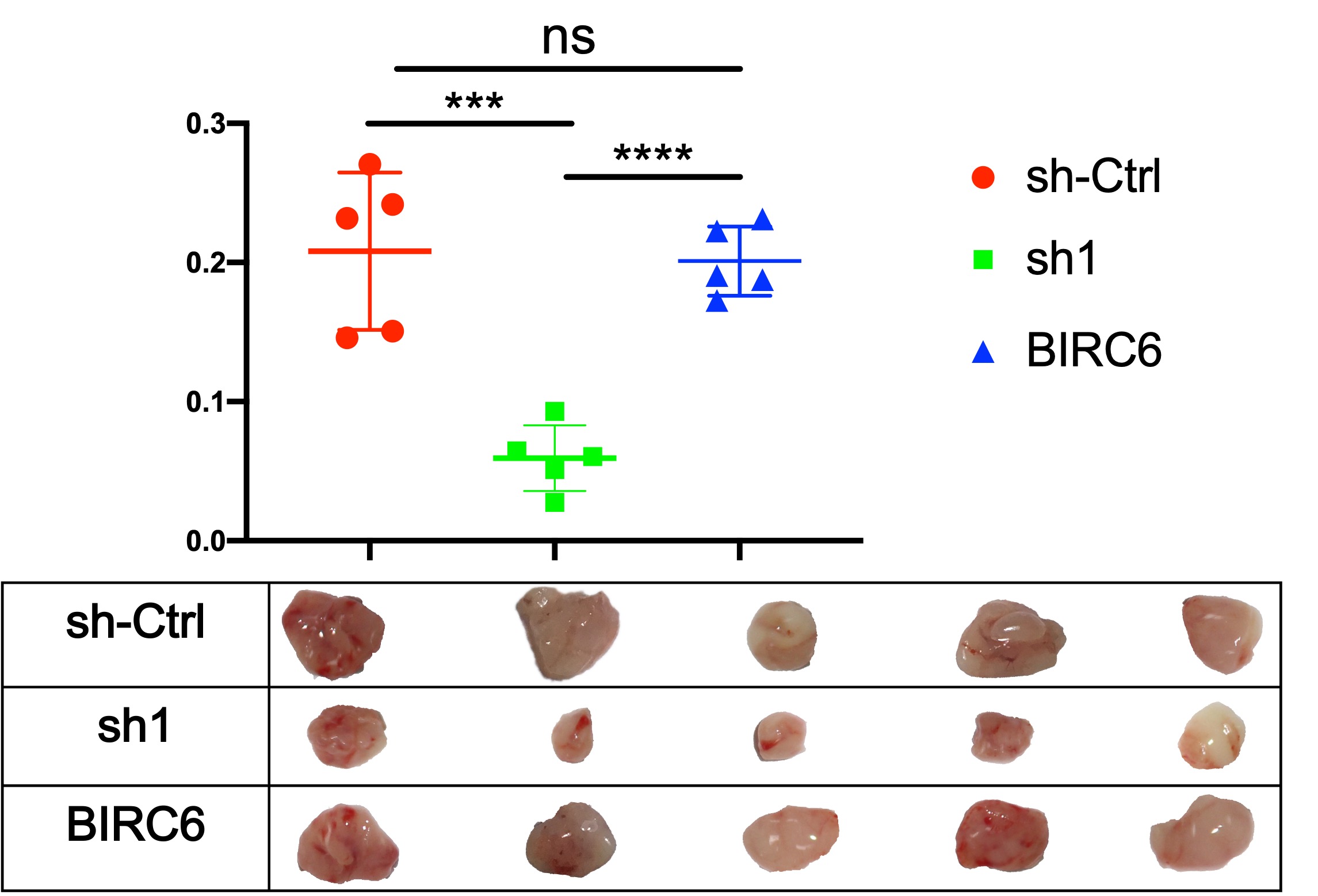

Supplement: Supplementary file 9 [file Image6.JPEG]
