# Supplementary material for: Identification of an autophagy-related 12-lncRNA signature and evaluation of NFYC-AS1 as a pro-cancer factor in lung adenocarcinoma
Source: Front Genet. 2022 Aug 11;13:834935. doi: 10.3389/fgene.2022.834935 (PMC9466988; doi:10.3389/fgene.2022.834935)
Supplement: Supplementary file 8 [file DataSheet1.DOCX]

FIG. 1. Flowchart of construction and validation of lncRNA risk signature and nomogram incorporating clinical parameters.

FIG. 2. Differently expressed autophagy related genes and lncRNAs based on the correlation coefficient | log2（FC）|≥0.5 and adjusted *p* < 0.05. (A) Volcano plot of differently expressed autophagy related genes. (B) Volcano plot of differently expressed lncRNAs between LUAD tumor and normal samples. FC, fold change.

Table. 1. Clinicopathologic Characteristics of LUAD Patients in primary and validation Cohorts

FIG. 3. Predictive power of the differently expressed lncRNAs in LUAD patients. (A) Univariate Cox regression analysis of differently expressed autophagy related lncRNAs. Cox analysis. (B) Multivariate Cox analysis of differently expressed autophagy related lncRNAs.

FIG. 4. The Kaplan–Meier survival analysis of 12 screened lncRNAs.

FIG. 5. The interaction network of autophagy genes and OS-associated lncRNAs in LUAD patients. (A) The co-expression network of the autophagy-related mRNAs and lncRNAs was constructed and visualized using Cytoscape. (B) The co-expression network with risk type information was visualized using Sankey diagram.

FIG. 6. Autophagy-related lncRNA risk score analysis of LUAD patients. (A) Top left, Kaplan-Meier survival analysis of the high-risk and low risk groups based on the median of risk score. Top right, 1-3-5-year AUC value of ROC analysis. Middle, Heatmap of autophagy related lncRNAs expression profiles in the low-risk and high-risk groups. Bottom, the scatterplot based on the survival status of each patient. (B) Similar results as 6A from TCGA train dataset. (C) Similar results as 6A from GSE validation dataset. (D) Nomogram of 12-lncRNA signature based on multivariate Cox proportional hazards regression analyses.

FIG. 7. Independent prognostic ability evaluation for the constructed prognostic signature in

LUAD. (A) Univariate Cox regression analysis of risk score and related clinical information regarding prognostic value in TCGA LUAD dataset. (B) Multivariate Cox analysis of risk model score and clinical features regarding prognostic value in TCGA LUAD dataset. (C) Univariate Cox regression analysis of risk score and related clinical information regarding prognostic value in GSE dataset. (D) Multivariate Cox analysis of risk model score and clinical features regarding prognostic value in GSE dataset.

FIG. 8. The 1-, 3-, and 5-year ROC curves of risk score and related clinical information of LUAD patients

FIG. 9. Nomogram incorporating risk score and other clinical information. Top, nomograms to predict 3-year or 5-year OS of patients with LUAD. Bottom, calibration curves of nomogram for 3-year or 5-year OS prediction.

FIG. 10. Quantitative reverse transcription polymerase reaction results of four lncRNAs, including HCG18, TMPO-AS1, NFYC-AS1 and LNC00996, from ten LUAD patients.

FIG. 11. Immunohistochemistry results of 34 pair of tissues from LUAD patients. (A) Representative results of BIRC6 expression in normal and tumor tissues based on the median of NFYC-AS1 expression level. (B) The quantitative scores of above IHC results. (C) BIRC6 expressions from TCGA database based on the median of NFYC-AS1 expression level.

FIG. 12. The influence of NFYC-AS1 and BIRC6 on the proliferation of LUAD cell lines. (A) RT-PCR results of NFYC-AS1 expression in A549 cells with or without shRNA knockdown. (B) The cell proliferation of A549 cells with or without knockdown of NFYC-AS1 expression. (C) Immunoblot of BIRC6 expression in A549 cells with or without knockdown of NFYC-AS1 expression. (D) The cell proliferation of A549 cells with knockdown of NFYC-AS1 expression or the rescue of BIRC6 expression.

FIG. 13. The influence of NFYC-AS1 on the autophagy of A549 cells. (A) Immunofluorescence analysis of key autophagy marker LC3B in A549 cells with or without knockdown of NFYC-AS1 expression. The white arrow indicates the significant expression of shRNA plasmids, whereas the red arrow indicates a less significant expression. (B) Immunoblot of Beclin 1 and SQSTM1/p62 expression in A549 cells with or without knockdown of NFYC-AS1 expression.

FIG. 14. The influence of NFYC-AS1 on the apoptosis of A549 cells. (A) Representative flow cytometry analysis of apoptosis in A549 cells without the knockdown of NFYC-AS1 expression. (B) Representative flow cytometry analysis of apoptosis in A549 cells with the knockdown of NFYC-AS1 expression. (C) Representative flow cytometry analysis of apoptosis in A549 cells with the knockdown of NFYC-AS1 expression combining with the rescue of BIRC6 expression. (D) Statistic results of apoptosis rate in A549 cells with or without the knockdown of NFYC-AS1 expression or combining the NFYC-AS1 knockdown with BIRC6 overexpression.

Supplementary Figure S1. HCG18, TMPO-AS1, NFYC-AS1 and LNC00996 expressions between normal and tumor tissues from TCGA LUAD database.

Supplementary Figure S2. HCG18, TMPO-AS1, NFYC-AS1 and LNC00996 expressions between normal and tumor tissues from GSE40791 datasets. G1 indicates normal and G2 indicates tumor tissues.

Supplementary Figure S3. The co-expression correlation analysis between OS-associated lncRNAs NFYC-AS1 and screened autophagy genes in LUAD patients. (A) The co-expression correlation analysis of NFYC-AS1 and 16 differentially expressed autophagy related genes was visualized using the “ggplot2” package of R software. (B) The gene expression correlation analysis of NFYC-AS1 and BIRC6 from TCGA LUAD dataset was performed using GEPIA. (C) The gene expression correlation analysis of NFYC-AS1 and BIRC6 from GTEx lung dataset was performed using GEPIA.

Supplementary Figure S4. Possible relationships between NFYC-AS1 and BIRC6. (A) Representative scoring grades for IHC results. (B) Overall expression level of BIRC6 between normal and tumor tissues from 34 LUAD patients. (C) Overall expression level of BIRC6 between normal and tumor tissues from TCGA LUAD database. (D) BIRC6 expression from in vivo tumor xenograft of nude mice between control and NFYC-AS1 knockdown groups.

Supplementary Figure S5. In vivo tumor xenograft experiment in nude mice showed that knockdown of NFYC-AS1 exhibit the inhibitory effect on tumor growth, whereas the overexpression BIRC6 could restore the tumor growth in nude mice.
